# Expansion on Stromal Cells Preserves the Undifferentiated State of Human Hematopoietic Stem Cells Despite Compromised Reconstitution Ability

**DOI:** 10.1371/journal.pone.0053912

**Published:** 2013-01-16

**Authors:** Mattias Magnusson, Maria I. Sierra, Rajkumar Sasidharan, Sacha L. Prashad, Melissa Romero, Pamela Saarikoski, Ben Van Handel, Andy Huang, Xinmin Li, Hanna K. A. Mikkola

**Affiliations:** 1 Department of Molecular, Cell and Developmental Biology, University of California Los Angeles, Los Angeles, California, United States of America; 2 Department of Pathology and Laboratory Medicine, University of California Los Angeles, Los Angeles, California, United States of America; 3 Eli and Edythe Broad Center for Regenerative Medicine and Stem Cell Research, University of California Los Angeles, Los Angeles, California, United States of America; 4 Jonsson Comprehensive Cancer Center, University of California Los Angeles, Los Angeles, California, United States of America; 5 Molecular Biology Institute, University of California Los Angeles, Los Angeles, California, United States of America; French Blood Institute, France

## Abstract

Lack of HLA-matched hematopoietic stem cells (HSC) limits the number of patients with life-threatening blood disorders that can be treated by HSC transplantation. So far, insufficient understanding of the regulatory mechanisms governing human HSC has precluded the development of effective protocols for culturing HSC for therapeutic use and molecular studies. We defined a culture system using OP9M2 mesenchymal stem cell (MSC) stroma that protects human hematopoietic stem/progenitor cells (HSPC) from differentiation and apoptosis. In addition, it facilitates a dramatic expansion of multipotent progenitors that retain the immunophenotype (CD34+CD38−CD90+) characteristic of human HSPC and proliferative potential over several weeks in culture. In contrast, transplantable HSC could be maintained, but not significantly expanded, during 2-week culture. Temporal analysis of the transcriptome of the *ex vivo* expanded CD34+CD38−CD90+ cells documented remarkable stability of most transcriptional regulators known to govern the undifferentiated HSC state. Nevertheless, it revealed dynamic fluctuations in transcriptional programs that associate with HSC behavior and may compromise HSC function, such as dysregulation of *PBX1* regulated genetic networks. This culture system serves now as a platform for modeling human multilineage hematopoietic stem/progenitor cell hierarchy and studying the complex regulation of HSC identity and function required for successful *ex vivo* expansion of transplantable HSC.

## Introduction

Hematopoietic stem cells (HSC) have been successfully used to treat leukemias, inherited immune deficiencies and other life-threatening blood diseases [1], [2]. However, only a fraction of patients benefit from this therapy due to the lack of HLA-matched bone marrow donors, and low number of HSC in cord blood [3]. Therefore, a long-standing goal has been to establish culture protocols to facilitate HSC expansion. However, there has been little success in expanding human HSC for clinical purposes due to limited understanding of the complex mechanisms governing HSC properties, and how these programs become compromised in culture. Furthermore, most HSC regulators have been identified using gene-targeted mouse models [4], whereas mechanistic understanding of human hematopoiesis is lagging behind due to lack of suitable *in vitro* and *in vivo* model systems for manipulating human HSC or their niche. A major challenge in culturing HSC is the difficulty to recreate the specialized microenvironment that regulates self-renewal of HSC within hematopoietic tissues; as a result, cultured HSC are subjected to rapid differentiation or death [5].

The bone marrow HSC niche consists of multiple cell types, including mesenchymal stem cells (MSC), osteoblasts, adipocytes, endothelial cells and macrophages [6], [7], [8], [9], [10]. The microenvironment directs HSC fate decisions by mediating cell-cell interactions and secreting soluble growth factors [8], [11], [12]. Although several HSC supportive cytokines (e.g. SCF, IL-11, IL-3, FLT-3, TPO, angiopoietin-like proteins, and the Notch1 ligand Dl1) [13], [14], [15], [16], cell-intrinsic stimulators of HSC expansion (e.g. HOXB4) [16], [17], [18] and inhibitors of negative HSC regulators (e.g. AhR signaling [19]) have been identified, these have not yet led to the establishment of routine clinical protocols for HSC expansion. Several studies have assessed the suitability of various stromal cell lines from fetal and adult hematopoietic tissues to support murine and human hematopoiesis [20], [21], [22], [23], [24], [25]; nevertheless, there has been little progress in expanding functional human HSC on these stroma lines. It is unclear to what extent the different HSC properties can be maintained in culture, and what molecular defects prevent robust expansion of transplantable HSC. Understanding how the *ex vivo* culture *per se* affects HSC function and molecular properties will be a critical step toward improving culture conditions for the expansion of HSC for clinical purposes, and also for the long-term goal to generate transplantable HSC in culture from human pluripotent stem cells.

To understand the behavior of human hematopoietic stem/progenitor cells (HSPC) in culture we established an MSC stroma based co-culture system for modeling human hematopoietic hierarchy, and defined the extent to which surface markers, functional properties and transcriptome characteristic for the primitive HSPC fraction can be preserved during culture. We show that OP9M2, a subclone of OP9 stroma cells, protects human fetal liver and cord blood HSPC from differentiation and apoptosis, facilitating a dramatic *ex vivo* expansion of multipotent hematopoietic cells that preserve the CD34+CD38−CD90+ surface immunophenotype that is characteristic for human HSC. This system also maintains the initial number of transplantable human fetal liver HSC (defined based on myelo-lymphoid reconstitution in NSG mice) for at least 2 weeks in culture, but does not support their significant expansion. Genome-wide gene expression analysis of the expanded fetal liver CD34+CD38−CD90+ cells showed a remarkably stable transcription factor network associated with HSC entity, but revealed dynamic changes in distinct molecular programs that are sufficient to compromise HSC function. Thus, this co-culture offers a robust *ex vivo* system for studying the regulation of human multilineage hematopoiesis. Furthermore, the temporal gene expression data from *in vivo* derived and *ex vivo* expanded human CD34+CD38−CD90+ will serve as a resource to identify key regulatory mechanisms that control HSC identity *vs.* function, and to develop clinically applicable protocols for HSC expansion and *de novo* generation from pluripotent stem cells.

## Materials and Methods

### Ethical Statement

This Study was carried out in strict accordance with the recommendations in the Guide for the Care and Use of Laboratory Animals of National Institutes of Health. The protocol was reviewed and approved by UCLA Animal Research Committee (Protocol number 2005–109). All efforts were made to minimize suffering.

This work does not involve research on human subjects based on the federal legislation (45 CFR 46.102(f)). Fetal livers were discarded material from elective terminations performed by Family Planning Associates. All material was obtained only after written informed consent and carried no personal identifiers. The consent form was approved by Family Planning Associates. The project was evaluated by the UCLA Medical Institutional Board 2 who determined that no IRB approval is required.

### Isolation of HSPC from Human Fetal Liver

Fetal livers were discarded material from elective terminations of second trimester pregnancies (14–18 weeks of developmental age). Single cell suspension was prepared by dissociation using scalpels and syringes. Red cells were removed using Ficoll gradient (Stem Cell Technologies). CD34+ cells were isolated using magnetic beads (Miltenyi Biotech).

### Stromal Cell Lines

OP9M2 subclone was generated by deriving a clonal line from OP9 stromal cells [26] plated at low density (0.32 cells/cm^2^) in stroma media containing α-MEM (GIBCO/Invitrogen), 20% fetal bovine serum (Hyclone) and 1% penicillin/streptomycin (GIBCO/Invitrogen). The BFC012 and AFT024 mouse fetal liver stromal lines was kindly provided by Kateri A Moore and Ihor Lemischka (Mount Sinai School of Medicine, NYC) [27]. S17 [28] (obtained from Kenneth Dorshkind at UCLA) and the MS5 [29] mouse bone marrow stromal lines were also tested.

### Hematopoietic Stem/progenitor Cell Cultures

Stromal cells were irradiated (2000 rad) and plated (25,000 cells/cm^2^) on tissue culture treated wells in stroma media (see above) 24 hours before co-culture. HSPC were co-cultured with stroma (1000–5000 CD34+ cells/cm^2^) in “HSC media” (stroma media supplemented with human SCF (25 ng/ml), FLT-3L (25 ng/ml) and TPO (25 ng/ml) (Peprotech) for 7–14 days before replating (100,000–200,000 cells/cm^2^). Half the media was replaced every other day. For B-cell differentiation, the expanded cells were co-cultured on irradiated OP9M2 in stroma media supplemented with human SCF (50 ng/ml), FLT-3L (40 ng/ml) and IL-7 (100 ng/ml) (Peprotech). Following expansion, cells were assayed by FACS and colony-forming assays (see below).

### Flow Cytometry

Mouse anti–human monoclonal antibodies for CD45, CD34, CD90, CD66b, CD235, CD13, CD14 and CD33 (BD Biosciences) and CD45, CD19, CD38 (eBioscience) were used for flow cytometry. Dead cells were excluded with 7-amino-actinomycin D (BD Biosciences). Cells were assayed on a BD LSRII flow cytometer and data was analyzed with FlowJo software (TreeStar). Cell sortings were performed on a BD Aria II.

### Colony-forming Assays

Single-cell suspensions were obtained as described and plated on MethoCult GF+ H4435 methylcellulose (Stem Cell Technologies) containing SCF, GM-CSF, IL-3 and EPO supplemented with TPO (10 ng/ml, Peprotech), 1% penicillin-streptomycin and 1% amphotericin B (GIBCO/Invitrogen). Colonies were scored after 14 days.

### Transplantation Assays

Transplantation assays were performed by intravenous (tail vein or retro-orbital) injection into sublethally (325 rad) irradiated 7–10 week female NOD-*scid IL2R*γ *null* mice (Jackson Laboratories) housed under pathogen-free conditions. For culture time course analysis, 50,000 CD34+ fetal liver cells or their expansion equivalent were transplanted. Peripheral blood was analyzed for human engraftment. Mice were sacrificed by CO_2_ 16 weeks post transplantation and bone marrow and spleen were analyzed for surface markers expressed in human HSPC and their differentiated progeny. The NSG repopulating cell (NSG-RC) frequency was determined using 5000, 1500 and 500 CD34+ fetal liver cells and their expansion equivalent after 2 weeks of co-culture on OP9M2. The cell dose was considered to contain at least one NSG-RC if human engraftment in the bone marrow exceeded 0.1%. The NSG-RC frequency was calculated on the basis of negative recipients using L-calc software (Stem Cell Technologies).

### RNA Purification and Microarray

RNA was purified from 100,000 CD45+CD34+CD38−CD90+ fetal liver cells that were isolated freshly (day 0) or at different time points in culture (12 hours, 2 weeks and 5 weeks) using QIAshredder and RNEasy Mini Kit (QIAGEN). The RNA was amplified using the NuGen FFPE amplification kit (Roche) and hybridized on Affymetrix arrays (u133plus2.0 array) in the Clinical Microarray Core, Department of Pathology & Laboratory Medicine at UCLA. For the OP9M2 and BFC012 stromal cell lines the RNA was amplified using an Ambion kit and hybridized to Affymetrix mouse 430 2.0 array. Raw data will be available for download from Gene Expression Omnibus (http://ncbi.nlm.nih.gov/geo) (GSE34974).

### Differential Gene Expression Analysis

Bioconductor version 2.7 was used for computational analyses [30], [31]. The samples were clustered using hierarchical clustering using Spearman rank correlation as the distance metric. Cluster 3.0 (for clustering) and Treeview (for visualization) were used. Limma package was used to obtain differentially expressed genes. The MAS5.0 algorithm through the R package Affy was used for calculating P/M/A detection calls for each array sample. The online resource DAVID was used to determine Gene Ontology (GO) statistically over-represented categories. The cellular location of the differentially expressed genes was annotated using Ingenuity Pathway Analysis software. Fuzzy c-means clustering was performed on microarray data from Day 0, 12 hours, 2 weeks and 5 weeks using Mfuzz. The probe sets included in the clustering were differentially expressed in at least one pair wise comparison at 5% FDR, and a fold change value of ±2.0. RMA derived expression values were standardized and clustered using Mfuzz for the probe sets that met the above criteria.

## Results and Discussion

### OP9M2 Stroma Supports Expansion of Human CD34+CD38−CD90+ Cells and Establishment of Multilineage Hematopoietic Hierarchy *ex vivo*

**Figure 1:**
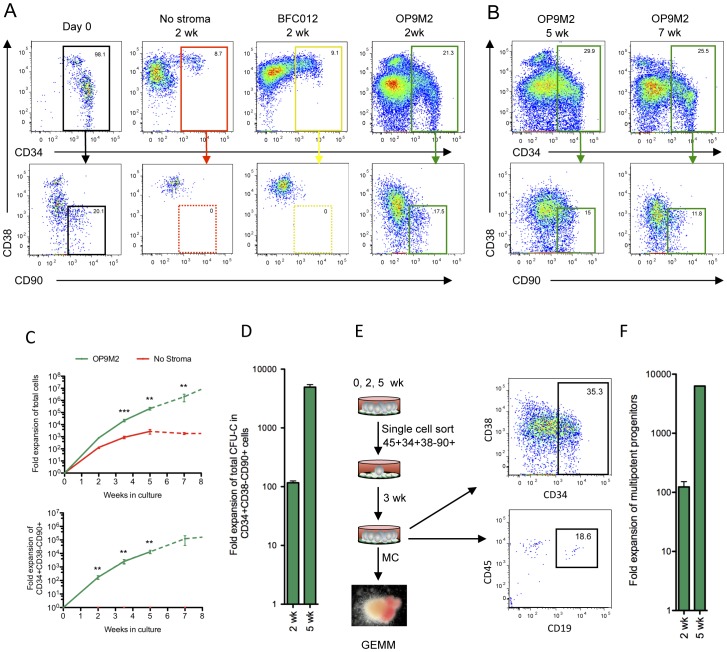
The OP9M2 MSC stromal cell line supports long-term expansion of multipotent human CD34+CD38 −**CD90+ cells.** Co-culture of human fetal liver CD34+ cells on OP9M2 stroma maintains cells with the characteristic CD34+CD38−CD90+ surface immunophenotype that HSC express. (A) Representative FACS plots of CD34+ fetal liver cells after co-culture either without stroma, on BFC012 stromal cells or on OP9M2 stromal cells are shown. (B) Representative FACS plots of fetal liver CD34+ cells co-cultured for 5 and 7 weeks on the OP9M2 stromal cell line are shown. (C) Upper chart shows total fold expansion of all hematopoietic cells from input CD34+ cells with or without OP9M2. Lower chart shows total fold expansion of CD34+CD38−CD90+ cells. Bold line represents 7 experiments (n = 7) while dashed lines represent 3 experiments (n = 3), as in some experiments HSPC cultured beyond 5 weeks had started to lose proliferative potential. (D) Fold expansion of CD34+CD38−CD90+ clonogenic progenitors (CFU-C, assessed in methylcellulose cultures) is shown. Error bars represent SEM (* p<0.05, n = 3). (E) Single cell assay documenting expansion of CD34+CD38−CD90+ cells that retain both myeloid and lymphoid differentiation potential is shown. 96-well plates were coated with OP9M2 stroma, and individual CD34+CD38−CD90+ fetal liver cells (freshly isolated or *ex vivo* expanded) were sorted directly into each well and cultured for 3 weeks to assess for recreating the CD34+CD38−CD90+ immature population and for myelo-lymphoid differentiation potential. (F) Total fold expansion of clonally multipotent progenitors in co-culture with OP9M2 is shown. Error bars represent SEM (* p<0.05, 2 weeks n = 3, and 5 weeks n = 1).

One of the long-standing goals in the field has been to establish a culture system that maintains HSC properties. In the absence of such system, the ability to model human hematopoiesis *in vitro* has been limited, and little progress has been made in expanding transplantable human HSC for clinical applications. Moreover, in order to succeed in the generation of HSC from human pluripotent stem cells, we have to understand how culture affects HSC properties, and minimize the negative effects of culture. Therefore, it is crucial to develop culture systems that mimic the *in vivo* HSC niche, as well as to understand the molecular changes that HSC acquire during culture. To define the extent to which HSC properties can be maintained in culture, we first assessed the ability of different stromal cell lines to preserve the undifferentiated state of human HSC (Figure 1A, Figure S1 and data not shown). We chose second trimester fetal liver HSC as the primary cell type for this study, as they are highly self-renewing, similar to cord blood, and also represent a developmentally closer target cell for generation of HSC from human ES cells or IPS cells. Culture of CD34+ human fetal liver cells with standard “HSC cytokines” (SCF, TPO and FLT-3L) with no stroma for two weeks resulted in loss of the primitive CD34+CD38−CD90+ population that contains HSC (Figure 1A). Likewise, the mouse BFC012 fetal liver stroma line and most of the mouse and human stroma lines tested resulted in complete loss or decrease of undifferentiated CD34+CD38−CD90+ cells during the two week culture (Figure 1A, Figure S1). In contrast, co-culture of CD34+ cells on OP9M2, a subclone of OP9 stroma maintained a robust population of CD34+CD38−CD90+ cells (Figure 1A). The parental OP9 also showed maintenance of CD34+CD38−CD90+ cells, albeit at slightly lower frequency (Figure S1). As the OP9M2 stroma is a clonal line and therefore more homogenous than the parental OP9 line, it was chosen for subsequent studies. Co-culture on OP9M2 cells also facilitated the generation of mature myeloid and erythroid cells, as well as B-cells if the culture was supplemented with IL-7 (Figure S2). These findings demonstrate that the OP9M2 stromal line maintains a population of human CD34+CD38−CD90+ cells in an undifferentiated state while supporting the establishment of multilineage hematopoietic hierarchy *ex vivo*. We found that the supportive OP9M2 stroma has MSC characteristics and can generate osteoblasts, adipocytes and chondrocytes (Figure S3), as also reported for the OP9 bulk cells [32], which is intriguing since MSCs and their derivatives are also key components of the bone marrow HSC niche *in vivo* [6], [8], [9].

### Co-culture on OP9M2 Stroma Supports Sustained *ex vivo* Expansion of Multipotent Human CD34+CD38−CD90+ Cells

We next assessed the ability of OP9M2 MSC stroma to sustain expansion of human CD34+CD38−CD90+ hematopoietic cells over time. Strikingly, CD34+CD38−CD90+ cells could be maintained on OP9M2 in similar frequencies for over 5 weeks, resulting in >10,000-fold expansion of cells with the surface immunophenotype associated with undifferentiated hematopoietic cells (Figure 1A–C). By 7–8 weeks of culture, the expansion potential of CD34+CD38−CD90+ cells had started to diminish (Figure 1C). Fractionation of the cultured (2 and 5 weeks) cells based on their surface phenotype showed long-term proliferative potential and capacity to expand phenotypic HSC exclusively within the CD34+CD38−CD90+ fraction (Figure S4), verifying that also during culture, the CD34+CD38−CD90+ phenotype identifies the most undifferentiated cells. Similar to human fetal liver, cord blood CD34+CD38−CD90+ cells could also be robustly expanded on OP9M2 (Figure S5A–C). Likewise, CD34+CD38−CD90+ cells were the only cells in cord blood expansion culture that that could regenerate hematopoietic cells with undifferentiated phenotype and long-term proliferative potential (Figure S5D–F).

To investigate the functional potential of the *ex vivo* expanded CD34+CD38−CD90+ cells, their colony forming potential and differentiation ability was assessed. Although there was a slight drop in frequency of clonogenic progenitors over time (CFU-C, as assessed on methylcellulose culture) among fetal liver CD34+CD38−CD90+ cells after culture (Figure S6A) a 110-fold and a 4900-fold total expansion of CD34+CD38−CD90+ clonogenic progenitors was obtained during 2 and 5 weeks, respectively (Figure 1D). Similar expansion of CD34+CD38−CD90+ CFU-C (89-fold (±8)) was obtained during 2 weeks culture of cord blood cells (data not shown). In addition, the distribution of different colony types was maintained among the CD34+CD38−CD90+ cells expanded for 2 weeks, whereas by 5 wk, there was a slight decrease in GEMM colony frequency (Figure S6B). To assess multipotency of the expanded CD34+CD38−CD90+ cells at clonal level, CD34+CD38−CD90+ cells were isolated freshly or after 2 or 5-week culture and plated individually on OP9M2 (Figure 1E), after which the clones were split into myeloid- and B-lymphoid conditions. Likewise, there was a 2.3-fold decline in the frequency of clonally multipotent clonogenic cells among the CD34+CD38−CD90+ cells after 2 weeks in culture, (Figure S6C), nevertheless, a 100-fold expansion of clonally multipotent CD34+CD38−CD90+ cells was observed after 2 weeks (Figure 1F), and in one experiment, 8000-fold expansion after 5 weeks in culture. Importantly, the expanded CD34+CD38−CD90+ cells remained responsive to microenvironmental cues and differentiated rapidly upon removal from the supportive stroma (Figure S7).

These data demonstrate that the OP9M2 stroma supports long-term expansion of human HSPC that preserve key characteristics associated with HSC, including CD34+CD38−CD90+ surface immunophenotype, high proliferative potential and multipotency.

### Transplantable HSC can be Maintained on OP9M2 Stroma

**Figure 2:**
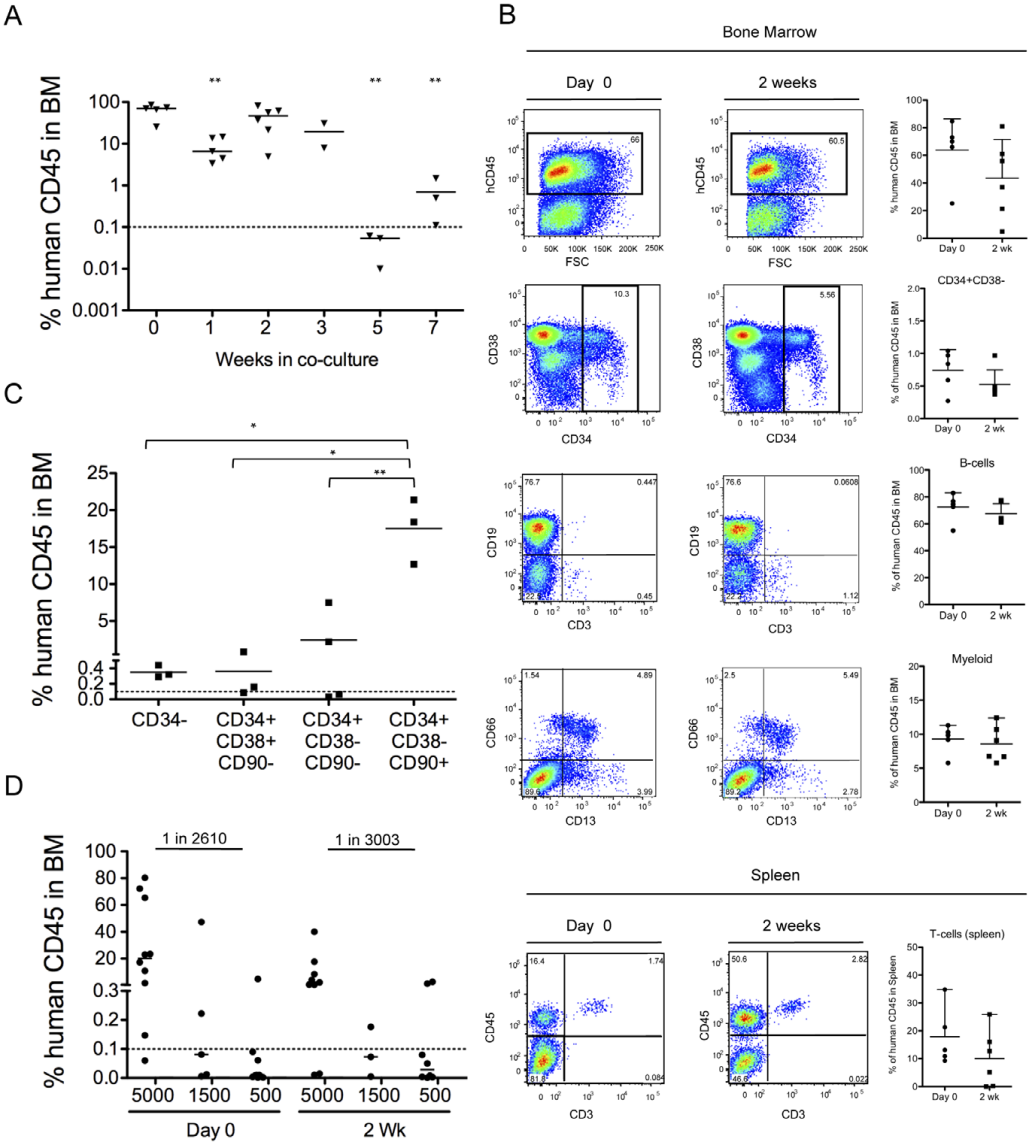
The OP9M2 stromal cell line supports maintenance of engraftable HSC. (A) Summary of total human reconstitution in the bone marrow of NSG mice 16 weeks post transplantation of all the progeny cells from 50,000 CD34+ input cells that were transplanted freshly (Day 0) or co-cultured for up to 7 weeks. Shown are all individual recipients and mean value. P value was calculated for each time-point in comparison to Day 0. (** p<0.01). (B) Representative FACS plot of bone marrow and spleen from NSG recipient mice showing long-term multilineage engraftment of 50,000 input CD34+ FL cells that were isolated freshly or co-cultured 2 weeks on OP9M2. (C) Comparison of engraftment capacity between purified CD34+CD38−CD90+, CD34+CD38−CD90-, CD34+CD38+CD90+ and CD34− cells 2 weeks after co-culture demonstrates that the engraftable HSC are maintained only in the CD34+CD38−CD90+ fraction. Each mouse was transplanted with the respective population derived from 50,000 input CD34+ human fetal liver cells in culture. Shown are all individual recipients and mean value from one experiment (** p<0.01, * p<0.05). (D) Limited dilution assays (n = 2) was performed to estimate NSG-RC frequency among fetal liver CD34+ cells that were transplanted freshly or after 2 week culture. Mice were transplanted with equal number of input CD34+ cells at different concentrations and analyzed 16 weeks post transplantation. No significant difference in NSG-RC number (Day 0∶1 in 2610, upper 4821, lower 1413 and 2 wk: 1 in 3003, upper 5953, lower 1515) (p = 0.77) was detected.

We next assessed the ability of the *ex vivo* expanded CD34+CD38−CD90+ cells to reconstitute the hematopoietic system *in vivo*. Transplantation of cells derived from 50,000 CD34+ fetal liver cells during 2-week culture into NSG mice demonstrated preservation of engraftable HSC on OP9M2 stroma. At 16 weeks post-transplantation, robust engraftment of human CD45+ cells was detected in both peripheral blood (2 wk: 14.55±8.51, Day 0∶29.93±7.67) and bone marrow (2 wk: 43.02±11.48, Day 0∶63.90±10.14) with contribution to undifferentiated CD34+CD38− cells, myeloid cells and B-cells (Figure 2A–B and Table S1). Furthermore, human T-cells were detected in the spleen (Figure 2B and Table S1). Multilineage human bone marrow engraftment was also observed from cells cultured for 3 weeks (18.26±12.35) (Table S1), whereas by 5 weeks in culture, their reconstitution ability had dropped dramatically (0.04%±0.015) (Figure 2A). Transplantation of *ex vivo* expanded cells fractionated into cells with the immunophenotype associated with undifferentiated HSPC (CD34+CD38−CD90+) and downstream progenitors (CD34+CD38−CD90-, CD34+CD38+CD90− and CD34−) confirmed that CD34+CD38−CD90+ cells in the cultured fetal liver possessed most of the *in vivo* HSC activity (Figure 2C and Table S1) as previously shown for cord blood HSC [33]. These results demonstrate that transplantable human HSC that retain the characteristic surface immunophenotype (CD34+CD38−CD90+) can be maintained *ex vivo* on OP9M2 stroma for over 2 weeks. Notably, previous work has suggested that CD38 is an unreliable differentiation marker in hematopoietic cells cultured *in vitro* [34]. However, this discrepancy could potentially be explained by different culture conditions, such as the lack of using OP9 MSC stromal cells. To investigate whether expansion of engraftable HSC was achieved, limiting dilution transplantation assay was performed. The number of NSG repopulating cells (NSG-RC) among cultured HSPC was similar to that before the expansion (Day 0∶1 in 2610 (lower 1413 and upper 4821) and at 2 wk in culture: 1 in 3003 (lower 1515 and upper 5953)) (Figure 2D and Table S1). As there was a dramatic expansion of CD34+CD38−CD90+ cells during culture (Figure 1C) but no increase in engraftable HSC, the frequency of NSR-RCs among CD34+CD38−CD90+ cells was estimated to drop from 1 in 522 to 1 in 63504. These data indicate that although HSC activity can be preserved during culture, majority of the expanded CD34+CD38−CD90+ cells fail to reconstitute the recipient’s hematopoietic system.

### Microarray Analysis of *ex vivo* Expanded CD34+CD38−CD90+ Cells Demonstrates Stable Expression of HSC Transcriptional Regulators

**Figure 3:**
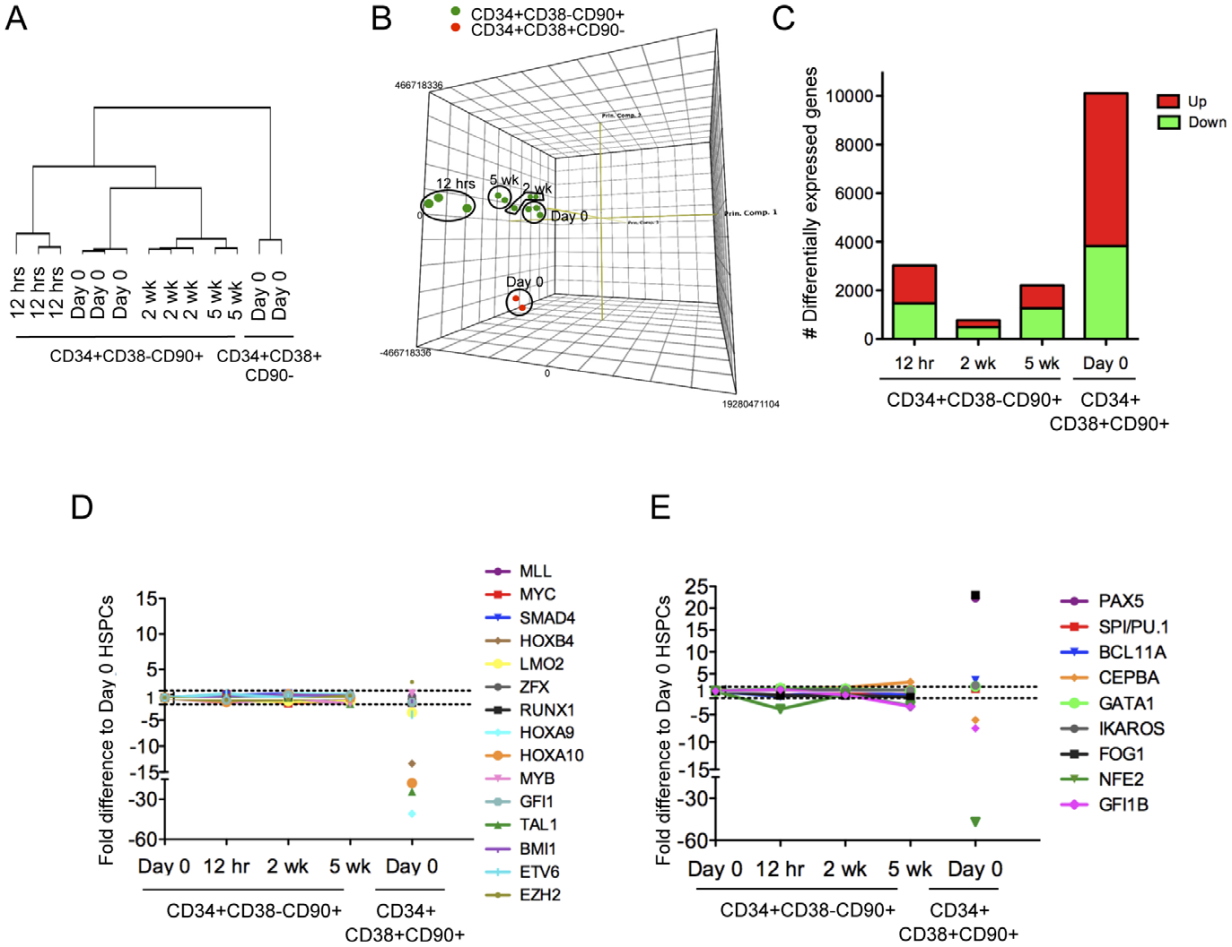
*Ex vivo* expanded CD34+CD38 −CD90+ cells demonstrate high preservation of the HSC transcriptional program. (A) Hierarchical clustering using spearman rank correlation and (B) principal component analysis comparing freshly isolated CD34+CD38−CD90+ cells (Day 0) to CD34+CD38+CD90− differentiated progenitors and to *ex-vivo* expanded CD34+CD38−CD90+ cells demonstrate high conservation of the HSC transcriptome during culture on OP9M2 stroma. (C) Number of genes with more than 2-fold change in gene expression compared to Day 0 CD34+CD38−CD90+ cells is shown. (D) Graph showing the relative expression of transcription factors known to regulate HSC development or maintenance. (E) Graph showing the relative expression of transcription factors known to initiate hematopoietic lineage commitment. Values represent a fold difference to freshly isolated CD34+CD38−CD90+ cells. Raw data is available for download from Gene Expression Omnibus (http://ncbi.nlm.nih.gov/geo) (GSE34974).

Despite the dramatic expansion of multipotent CD34+CD38−CD90+ cells on OP9M2 MSC stroma, there was no significant expansion of transplantable HSC, suggesting that most expanded CD34+CD38−CD90+ cells are functionally compromised. To define at the molecular level the degree to which “HSC transcriptional identity” can be preserved during culture and to identify the molecular blocks preventing successful expansion of fully functional HSC, we performed a genome-wide gene expression analysis of sorted CD34+CD38−CD90+ cells isolated at different stages of co-culture. To distinguish between molecular changes acquired immediately upon exposure to culture *versus* over prolonged culture, gene expression in sorted CD34+CD38−CD90+ cells was assessed after 12 hours, 2 weeks and 5 weeks in culture. Cultured CD34+CD38−CD90+ cells were compared to freshly isolated CD34+CD38−CD90+ cells and the more differentiated CD34+CD38+CD90- progenitors. Hierarchical clustering grouped all replicates together, demonstrating high reproducibility of gene expression in cultured CD34+CD38−CD90+ cells (Figure 3A). Spearman rank correlation and principal component analysis revealed that all cultured CD34+CD38−CD90+ cells were more similar to the freshly isolated CD34+CD38−CD90+ cells than the freshly isolated CD34+CD38+CD90- progenitors (Figure 3A–B), indicating that the transcriptome of the expanded CD34+CD38−CD90+ cells is highly preserved throughout the culture. Strikingly, CD34+CD38−CD90+ cells sorted after 2 weeks of co-culture were more similar to freshly isolated CD34+CD38−CD90+ cells than those cultured for only 12 hours (Figure 3A–B), suggesting that CD34+CD38−CD90+ cells have the capacity to adopt to their new stromal niche upon continued culture. However, the number of differentially expressed genes among CD34+CD38−CD90+ cells increased again substantially after prolonged culture (5 weeks) (Figure 3C).

We next investigated whether the expression of 33 transcriptional regulators that are known to be required for HSC development and/or maintenance were maintained in CD34+CD38−CD90+ cells sorted after culture. 26 of them (*SCL/TAL1, RUNX1, GFI1, BMI, HOXB4, HOXA9* etc.) remained stably expressed (<2-fold differentially expressed) throughout the entire 5-week culture (Figure 3D and Table S2). Strikingly, at 2 weeks in culture, *PBX1* was the only one of the known HSC transcription factors assessed that was downregulated more than 2-fold (p<0.05) (Table S2), documenting a remarkable stability of HSC regulators in *ex vivo* expanded CD34+CD38−CD90+ cells.

In line with these data, lineage specific transcription factors that are known to be crucial in initiating lymphoid, myeloid or megakaryocyte/erythroid linage differentiation (*PAX5, BCL11A, PU.1, CEBPα*, *NFE2* and *GATA1* etc. [35], [36], [37], [38] were not upregulated in CD34+CD38−CD90+ cells during 2 weeks of culture, and even by 5 weeks, only *CEBPα* was marginally activated (Figure 3E). This suggests that co-culture of human CD34+CD38−CD90+ cells on OP9M2 not only preserves a remarkably stable expression of HSC regulators but also suppresses the activation of canonical differentiation programs, thereby highly preserving the HSPC transcriptome during culture.

**Figure 4:**
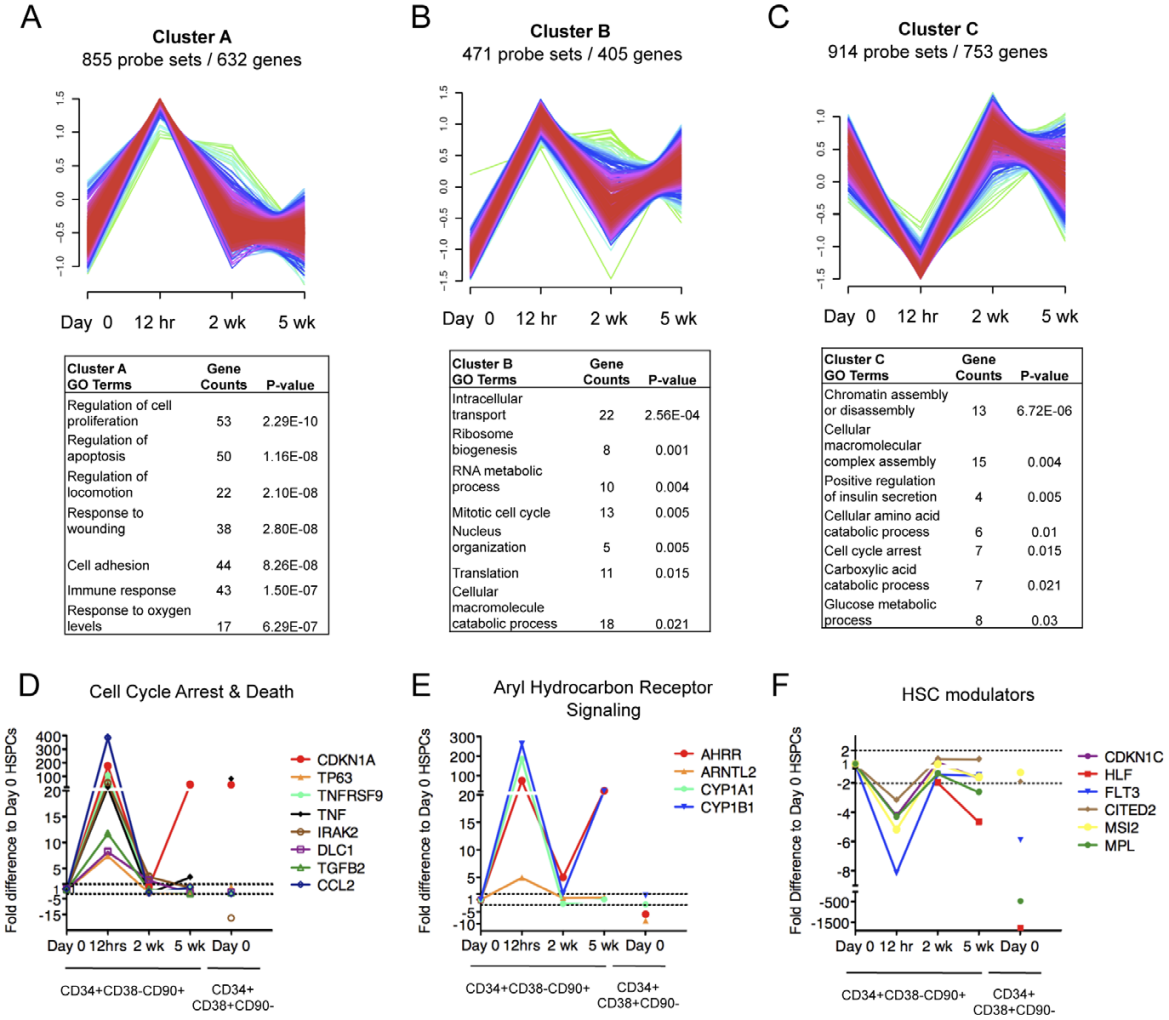
CD34+CD38−CD90+ cells display a transient “culture shock” upon exposure to culture. Fuzzy C cluster analysis identified three temporal gene expression patterns consisting of genes whose expression became transiently altered by 12 hrs in culture. (A) Cluster of genes upregulated at 12 hrs is shown together with their Gene ontology (GO) analysis. (B) Cluster of genes upregulated at 12 hrs and at 5 wk is shown with their GO analysis, (C) Cluster of genes downregulated at 12 hrs is shown with their GO analysis. (D) Transient upregulation of cell cycle arrest and cell death genes selected from clusters in A and B is shown. (E) Aryl hydrocarbon receptor signaling genes selected from cluster A are shown. (F) A temporary decrease in gene expression was observed in known HSC regulators; individual genes selected from cluster C are shown. Shown are fold differences in gene expression of *ex vivo* expanded CD34+CD38−CD90+ cells relative to freshly isolated (day 0) control CD34+CD38−CD90+ cells. CD34+CD38+CD90- indicates gene expression changes for differentiated progenitors relative to Day 0 control CD34+CD38−CD90+ cells.

**Figure 5:**
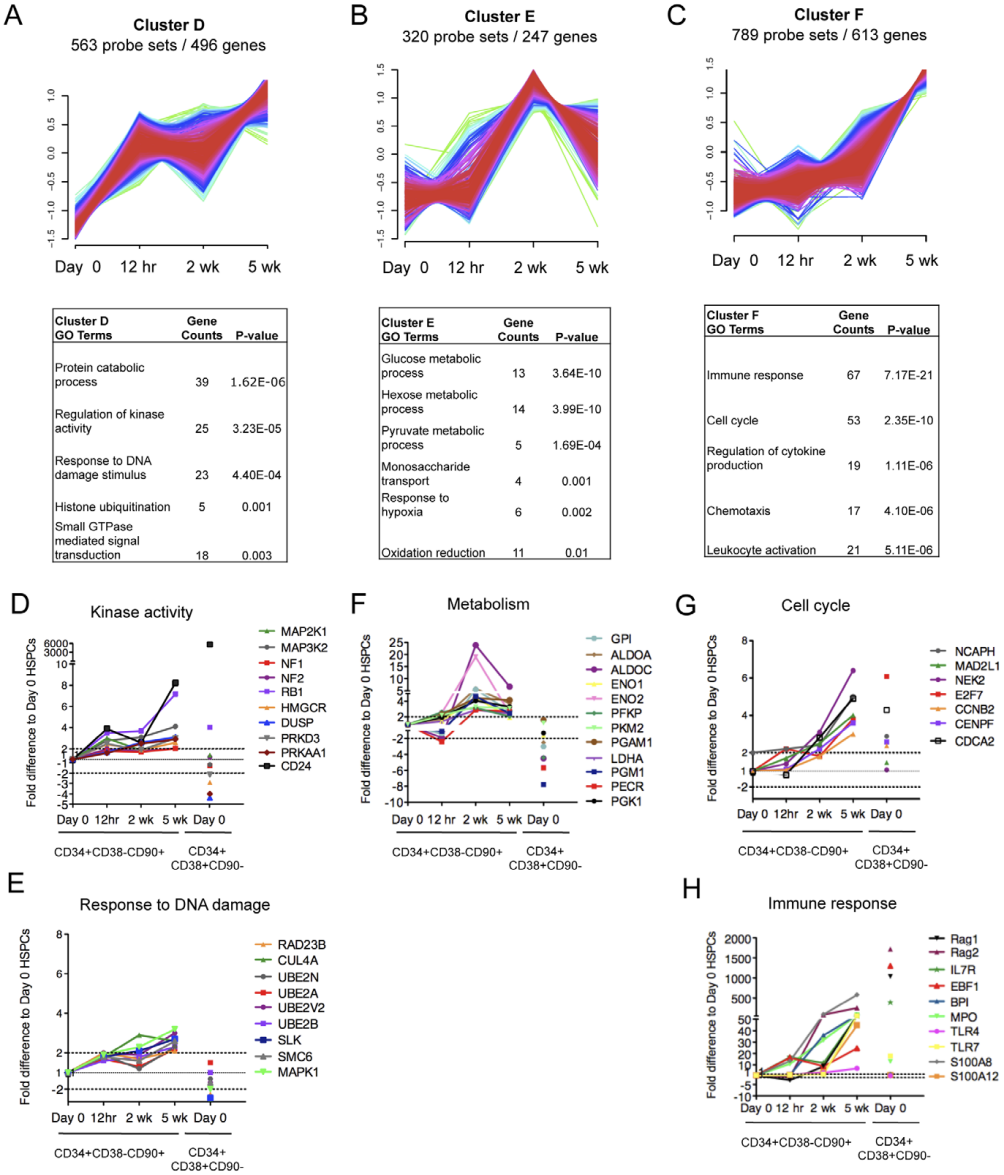
*Ex vivo* expanded CD34+CD38 −CD90+ cells show dynamic changes in transcriptional programs associated with cellular state. Fuzzy C cluster analysis identified three temporal gene expression patterns describing genes that were either upregulated (A) immediately upon culture, (B) predominantly at 2 weeks of culture or (C) gradually during prolonged culture. The GO analysis of genes in (A), (B) and (C) is shown below each cluster respectively. The GO analysis from cluster D identified several genes associated with (D) Kinase activity and (E) response to DNA damage that were modestly upregulated during culture. (F) GO analysis from cluster E identified several genes regulating glucose metabolism. GO analysis of cluster F identified several genes associated with (G) Cell cycle and (H) Immune response that were upregulated during prolonged culture. Shown are fold difference in gene expression of CD34+CD38−CD90+ cells at each time point relative to Day 0 control CD34+CD38−CD90+ cells. CD34+CD38+CD90- indicates gene expression changes for differentiated progenitors relative to Day 0 control CD34+CD38−CD90+ cells.

**Figure 6:**
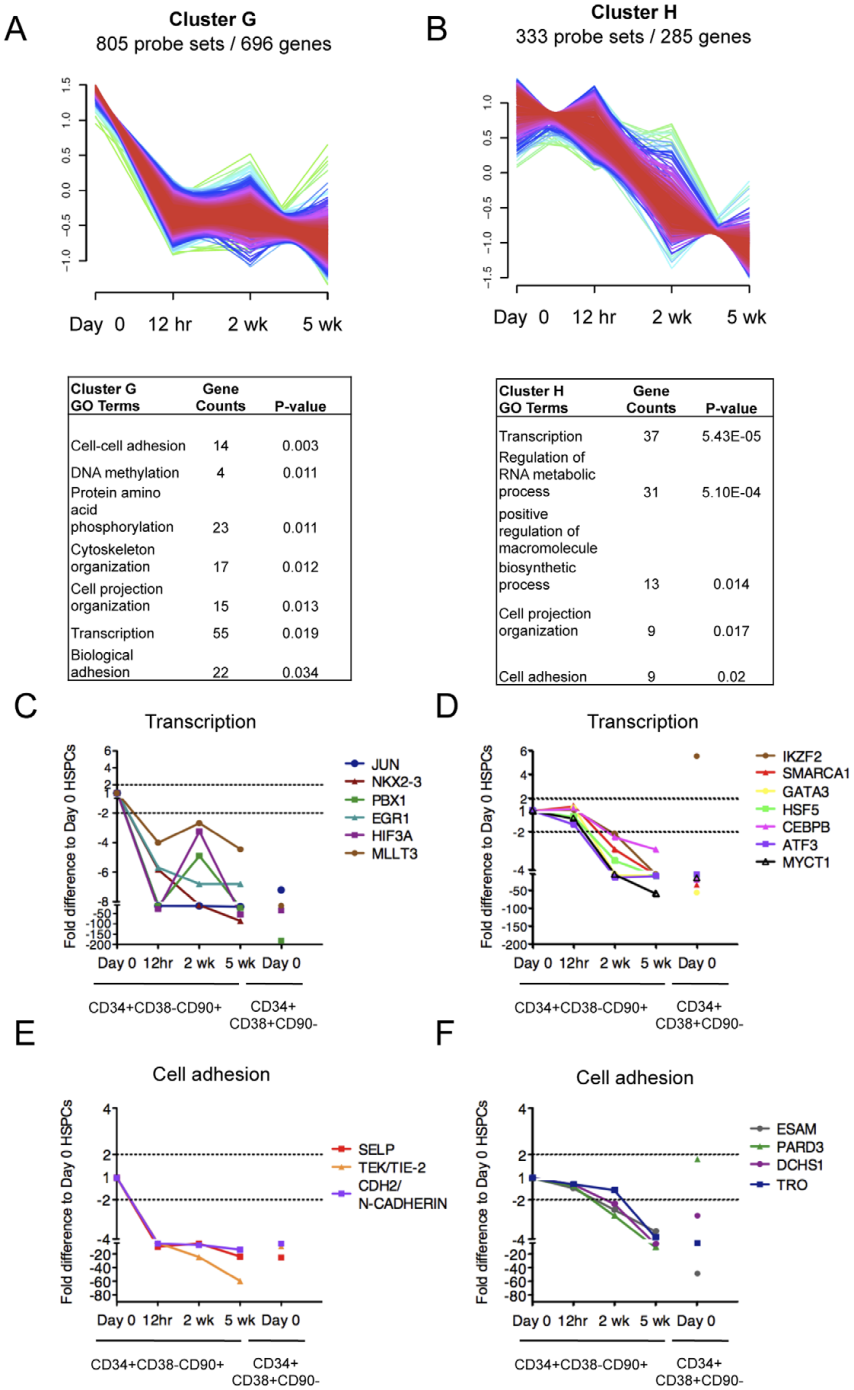
*Ex vivo* expanded CD34+CD38 −CD90+ cells show temporal changes in transcriptional programs associated with HSPC function and cell adhesion. Fuzzy C cluster analysis identified two temporal gene expression patterns describing genes that were either (A) immediately downregulated upon culture or (B) downregulated progressively during culture. GO analysis of genes in (A) and (B) (shown below each cluster respectively) identified numerous transcription factors that were downregulated, either (C) immediately or (D) progressively during culture. Similarly, several cell adhesion molecules implicated in HSC-niche interactions were downregulated either (E) immediately or (F) progressively during the culture. Shown are fold difference in gene expression of CD34+CD38−CD90+ cells at each time point relative to Day 0 control CD34+CD38−CD90+ cells. CD34+CD38+CD90− indicates gene expression changes for differentiated progenitors relative to Day 0 control CD34+CD38−CD90+ cells.

In order to understand the nature of the transcriptional changes in CD34+CD38−CD90+ cells sorted after culture, we analyzed the 3110 genes identified as >2-fold differentially expressed at least in one time point during the 5-week culture (Table S3). To identify patterns of co-regulation within the differentially expressed genes, a fuzzy c-means cluster analysis was performed. Eight distinct temporally changing gene expression patterns with unique GO categories were identified (Figure 4A–C, 5A–C and 6A–B). This analysis demonstrated that, despite their shared surface phenotype, stability of most known HSC transcription factors and ability to resist premature differentiation, the *ex vivo* expanded CD34+CD38−CD90+ cells undergo dynamic gene expression changes during culture.

### CD34+CD38−CD90+ Cells Display a Transient “Culture Shock” Upon Exposure to Culture

Interestingly, three clusters (A–C) contained genes whose expression changed already by 12 hours in culture, but was largely restored by 2 weeks (Figure 4A–C, Table S3). GO analysis of clusters A and B which showed a transient upregulation revealed enrichment for genes regulating cell proliferation and apoptosis (Figure 4A–B). Cluster A genes included *CDKN1A* (*p21*), *TP63* and members of the TNF signaling pathway, all known regulators of cell cycle arrest and cell death (Figure 4D) [39], [40], [41], suggesting that OP9M2 stroma can provide signals that protect the primitive CD34+CD38−CD90+ cells from apoptosis. Furthermore, several members of the aryl hydrocarbon receptor (AhR) signaling pathway (*AHRR*, *CYP1A1* and *CYP1B*), which has been recently identified as a negative regulator of HSC expansion [19], were upregulated at 12 hours, while at 2 weeks, the levels were remarkably similar to non-cultured CD34+CD38−CD90+ cells (Figure 4E). Analysis of published data of genes suppressed upon blocking AhR signaling in culture using SR1 (Stemregulin 1) [19] revealed that 9 out of 11 of the AhR signaling responsive genes became transiently induced in CD34+CD38−CD90+ cells on OP9M2 MSC stroma, but were a least temporarily restored during continued culture (Figure S8). These data indicate that the OP9M2 stroma can provide critical niche signals to suppress the AhR pathway that would negatively affect HSC function. Moreover, a transient decrease in gene expression was observed in other factors previously associated with HSC function such as *CDKN1C, HLF* and *FLT3* (Figure 4F) [42], [43], [44]. The immediate fluctuation in gene expression of negative and positive HSC regulators, followed by restoration to almost normal levels upon further culture suggests that cultured CD34+CD38−CD90+ cells experience an initial “culture shock” after which they are still capable of adapting to a new supportive niche on OP9M2 stroma with a transcriptome that highly resembles freshly isolated CD34+CD38−CD90+ cells.

### *Ex vivo* Culture of CD34+CD38−CD90+ Cells Induces Dynamic Changes in Cellular State

Clusters D, E and F contained genes that were upregulated at some point in culture (Figure 5A–C, Table S3). Cluster D genes that showed a trend for upregulation already by 12 hours and mostly stayed upregulated throughout the culture were enriched for genes involved in kinase activity and DNA repair (Figure 5D–E). However, the expression changes for genes in these categories were only modest, and mainly observed at 5 weeks of culture, providing little evidence for compromised genomic integrity in the expanded CD34+CD38−CD90+ cells or other explanation for their dysfunction.

Cluster E, which showed upregulation by 2 weeks of culture when the transcriptome of CD34+CD38−CD90+ cells was otherwise highly reminiscent of the freshly isolated CD34+CD38−CD90+ cells (Figure 5B), revealed alterations in metabolic genes. Many of these genes were involved in glucose metabolism and specifically in glycolysis (Figure 5F). This is intriguing, as HSC are known to utilize glycolytic metabolism rather than oxidative phosphorylation as their preferred energy metabolism [45]. Although it is not known whether increased expression of glycolytic genes would compromise HSC function, this data draws attention to the culture conditions (e.g. glucose content, oxygen tension etc.) that change significantly between the *in vivo* niches and *ex vivo* culture, and may impact HSC function.

Cluster F genes that were upregulated gradually during prolonged culture were enriched for genes involved in cell cycle regulation and immune response (Figure 5C, 5G–H). Notably, the expression of genes involved in cell cycle regulation (e.g. E2F7, which is induced by p53 upon genotoxic stress and negatively regulates cell cycle [46], and CCNB2 and NEK2 which accumulate during S phase and G2, etc.) was only marginally upregulated by 2 weeks when the CD34+CD38−CD90+ cells still possessed high proliferative potential, but increased by 5 weeks when the expansion potential of CD34+CD38−CD90+ cells had started to plateau (Figure 5G). Interestingly, specific genes normally expressed in mature myeloid (*MPO and BPI*) [47], [48], [49] or lymphoid cells (*IL7R, RAG1* and *RAG2*) [50], [51] as well as toll like receptors (TLR4 and TLR7) and their agonists, S100 proteins, were significantly upregulated already by 2 weeks in culture (Figure 5H). Importantly, induction of the inflammatory genes in CD34+CD38−CD90+ cells does not seem to reflect promiscuous activation of the canonical differentiation programs, as the key lineage specific transcription factors (e.g. *PAX5, BCL11A, PU.1*, *GATA1, NFE2* etc.) remained suppressed in CD34+CD38−CD90+ cells throughout the culture period (Figure 3E). The etiology and significance of the upregulation of these immune response genes is unknown, as MSCs have been shown to rather have immunosuppressive properties and even reduce the risk for graft failure when transplanted together with HSC [52], [53]. If the species mismatch between human HSPC and mouse stroma or fetal calf serum can elicit an immune response in cultured human CD34+CD38−CD90+ cells, it will be important to recreate similar the supportive culture conditions using non-animal products.

### *Ex vivo* Expansion Results in Downregulation of Transcription Factors and Adhesion Molecules in CD34+CD38−CD90+ Cells

Our findings indicate that even though *ex vivo* expanded CD34+CD38−CD90+ cells demonstrate dynamic changes in “cell state” (e.g. kinase activity, metabolism, cell cycle etc.), based on the transcriptional regulators that govern “cell identity” (e.g. undifferentiated HSC *vs.* lineage committed cell), they still maintain the identity of an HSC. As such, these data provided little clues to why the majority of the *ex vivo* expanded CD34+CD38−CD90+ cells do not function properly upon transplantation. However, the two remaining clusters of differentially expressed genes contained genes that were progressively downregulated during culture, either immediately by 12 hours (cluster G) or during prolonged culture (cluster H) (Figure 6A–B). Interestingly, both clusters were enriched for transcriptional regulators, including *PBX1* (cluster G) that is crucial for HSC function [54], *EGR1* (cluster G) that is required to maintain HSC in the niche although its loss does not compromise HSC function [55], and GATA3 (cluster H) (Figure 6C–D). GATA3 is required for proper T-cell development and implicated in the regulation of adult HSC function, although gata3 deficient HSC from neonatal mice or fetal liver demonstrated no functional defect [56], [57]. Moreover, cluster G and H genes that were downregulated during culture also included several surface proteins that have been implicated to participate in HSC-niche interactions, such as *TEK (TIE2)* and *N-CADHERIN* (Figure 6E) [11], [12], as well as molecules associated with cell projections, polarity, asymmetric division (*DCHS1* and *PARD3*) and implantation (TRO) [58], [59], [60] (Figure 6F). By 5 weeks, many of these genes had been downregulated to levels comparable to the more differentiated CD34+CD38+CD90- cells (Figure 6C–F). This indicates that, despite maintaining the characteristic HSC surface immunophenotype and most known HSC transcription factors as well as suppressing lineage differentiation, the expanded CD34+CD38−CD90+ cells have altered the expression of a unique subset of transcriptional regulators and cell surface proteins, which may impair their ability to fully maintain HSC function upon transplantation.

### Dysregulation of *PBX1* Compromises the Transcriptome of *ex vivo* Expanded CD34+CD38−CD90+ Cells

**Figure 7:**
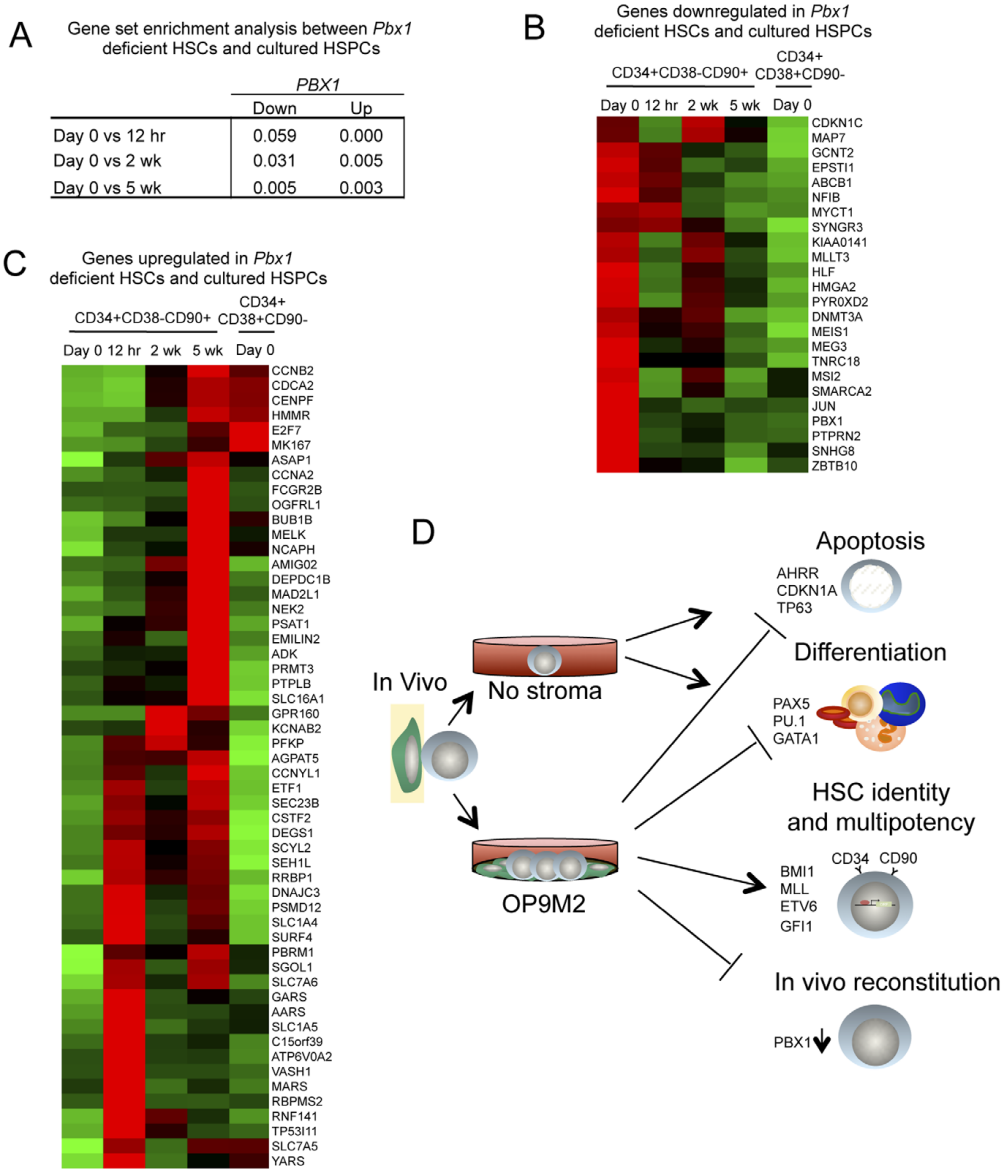
Cultured human CD34+CD38 −CD90+ cells show shared gene expression changes with *Pbx1* deficient mouse HSC. Differential gene expression analysis was performed comparing HSC (LSKCD34-Flt3-) from control and *Pbx1* deficient mice (GEO series GSE9188). (A) Table showing the FDR-q values from the GSEA analysis on pairwise expression data comparing freshly isolated CD34+CD38−CD90+ human cells with each time point in culture to the differentially expressed genes from *Pbx1* deficient mouse LSKCD34-Flt3- cells. (B) Heat map showing 24 genes downregulated both in Pbx1 deficient LSKCD34-Flt3- cells and at least in one of the culture time points (right) (>1.5-fold with respect to freshly isolated CD34+CD38−CD90+ cells, FDR of 5%). (C) Heat map of 54 genes that were upregulated both in *Pbx1* deficient HSC and at least in one of the culture time points (left), (>1.5-fold with respect to freshly isolated CD34+CD38−CD90+ cells, FDR of 5%). (D) A summary figure illustrating the HSC properties that are preserved or lost in the presence or absence of supportive stromal cells.

Our finding that the cultured CD34+CD38−CD90+ cells showed a remarkably stable expression of most known HSC transcription factors suggests that “HSC identity” *per se* is not lost upon *ex vivo* expansion. An exception to this was *PBX1*, which was downregulated already at 12 h and never fully recovered, indicating that *PBX1* expression is highly dependent on microenvironmental signals that were not recapitulated in culture. *Pbx1* deficiency in mice disrupts HSC self-renewal due to loss of stem cell quiescence and severely compromises their *in vivo* repopulation ability [54]. Therefore, we sought to define the extent to which the transcriptional changes in cultured CD34+CD38−CD90+ cells correlate with those dysregulated in *Pbx1* deficient mouse HSC. Indeed, gene set enrichment analysis (GSEA) showed a very high statistically significant correlation between *Pbx1* deficient mouse LSKCD34-Flt3- cells (Lin-Sca1+cKit+CD34-Flt3-) cells and cultured human CD34+CD38−CD90+ cells (Figure 7A). Direct comparison of the differentially expressed genes showed that 24 of the genes downregulated (>1.5-fold) and 54 genes upregulated in *Pbx1* deficient HSC (LSKCD34-Flt3-) showed similar dysregulation with respect to genes that changed in culture (Figure 7B–C). These included many downregulated transcriptional regulators, some of which are known to regulate hematopoiesis such as *MLLT3, MSI2, MEIS1, DNMT3A* and *HLF* [42], [61], [62], [63], [64] while others have so far not been associated with a clear function in HSPC biology (*MYCT1, HMGA2, SMARCA2* etc) (Figure 7B). The shared upregulated genes included many cell cycle- and mitosis genes *(NCAPH, NEK2, CCNB2, CDCA2* etc.) (Figure 5G and 7C). Although the specific function of many of the Pbx1 dependent gene in HSC still remains to be investigated, these data identify dysregulation of *PBX1* as a factor compromising the otherwise highly stable HSC transcriptional network in human CD34+CD38−CD90+ cells during expansion on MSC stroma, and nominate loss of *PBX1* as a possible defect contributing to the functional limitations in cultured HSPC (Figure 7D).

### Conclusion

We have defined a MSC co-culture system that expands clonally multipotent human hematopoietic cells that are protected from adverse effects that normally occur during culture (e.g. activation of aryl hydrocarbon receptor signaling, apoptosis, and immediate differentiation), and retain the CD34+CD38−CD90+ HSC surface immunophenotype and remarkably stable HSC transcription factor network for several weeks in culture. These data revealed that the identity of human HSC is highly preserved during *ex vivo* expansion on supportive MSC stroma despite the dynamic fluctuations in gene expression programs reflecting different cellular states (cell cycle, metabolism, etc). However, the fact that transplantable HSC could only be maintained, but not significantly expanded, during culture, indicated that most of the *ex vivo* expanded CD34+CD38−CD90+ cells are functionally compromised. Downregulation of *PBX1* was identified as a possible factor contributing to the dysregulated gene expression programs and the functional limitations in cultured CD34+CD38−CD90+ cells. It is important to note that the microarray analysis of cultured CD34+CD38−CD90+ cells was performed on human fetal liver cells, which are functionally different from HSC from adult bone marrow or cord blood as they have the highest self-renewal potential [65]. However, although fetal liver HSC are rarely used for clinical purposes due to ethical and legal considerations, they are developmentally the closest target for generation of HSC from ES or IPS cells, and therefore these data not only give important clues about the mechanisms regulating self-renewal but also how to improve the culture conditions for the generation of self-renewing HSC *de novo* from pluripotent cells.

This culture system serves now both as a useful platform for studying the regulation of human HSPC and multilineage hematopoietic hierarchy *ex vivo*, and as a starting point to develop protocols for the expansion or *de novo* generation of transplantable HSC for clinical applications. To achieve this goal, it will be critical to identify the signals that would help preserve the dysregulated HSC transcription factors identified in the study, such as *PBX1.* Notably, one report demonstrated that by knocking down *pbx1* in *HOXB4* over-expressing mouse HSC improved HSC expansion as compared to overexpression of *HOXB4* alone [66], although other studies had documented loss of HSC function upon knockdown of *pbx1* [54]. Notably, HOXB4 expression was not altered during our expansion culture. These data suggest that the effects of loss or overexpression of individual transcription factors may be context dependent, and also influenced by the time how long the gene is suppressed. Future overexpression and knockdown studies will be required to determine the degree to which *PBX1,* or the other transcription factors and signaling pathways dysregulated in culture, contribute to the loss of HSC function. However, as many of the transcription factors that were downregulated during culture are also putative oncogenes, their mere over-expression is unlikely to provide a clinically viable option for expanding functional HSC. Instead, future studies should focus on complementing the OP9M2 co-culture with other niche cells or extrinsic cues to better recapitulate the *in vivo* niche, such as Akt-activated endothelial cells, which express HSC supportive angiocrine factors IGFB2 and FGF2 [10] that are not expressed by OP9M2 stroma (data not shown). Moreover, as we also observed dynamic changes in HSPC metabolism during the culture, these findings suggest that optimization of the physiological parameters such as glucose and oxygen levels could further improve HSC function. Indeed, the HSC niche is hypoxic and culture of HSC in low oxygen levels has been reported to increase HSPC function [67], [68].

The fact that excessive proliferation of HSC *in vivo* in mice has been associated with decreased HSC function [69] inevitably raises the question whether the cell divisions in culture cause a reversible dysfunction in engraftment, or have already led to permanent loss of “stemness”. Nevertheless, our discovery of the high preservation of “HSC identity” in human CD34+CD38−CD90+ cells cultured on MSC stroma provides hope that by modulating the culture conditions to preserve, or even return, the *ex vivo* expanded CD34+CD38−CD90+ cells to the correct “cellular state” that allows efficient engraftment *in vivo* will take us closer to expanding functional HSC for the treatment of leukemia and other blood diseases.

## Supporting Information

Figure S1 **Co-culture of human CD34+ fetal liver on different stromal cell lines.** Representative FACS plots of CD34+ fetal liver cells after 2 weeks of co-culture on different mouse stroma cell lines. Presence of CD34+CD38−CD90+ cells indicates maintenance of undifferentiated human hematopoietic cells.
(TIF)

Figure S2 **The OP9M2 stroma line facilitates establishment of a multilineage human hematopoietic hierarchy** ***ex vivo*** **.** FACS analysis of CD34+ FL cells cultured on OP9M2 supplemented with SCF, TPO and FLT3L demonstrating, (A) forward and side scatter of cultured cells, (B and C) presence of HSPC (CD33) and various myeloid progenitors, monocytes and granulocytes (CD33, CD14, CD66 and CD13) as well as (D) erythroid cells (CD71). (E) CD34+ FL cells co-cultured on OP9M2 with IL-7, FLT-3L and SCF demonstrating robust generation of B-cells (CD19).
(TIF)

Figure S3 **The OP9M2 stroma line has mesenchymal stem cell properties.** OP9M2 stromal cells exhibit tri-lineage differentiation capacity into adipocytes, chondrocytes and osteoblasts. Adipocyte potential was determined by Oil Red O stain; chondrogenic potential by Safranin O stain; and osteocytic potential by Alizarin red stain. Images were acquired at 20× original magnification using an Axiovert 40CL microscope and a Canon Powershot G6 camera.
(TIF)

Figure S4 **Long-term proliferative potential and capacity to maintain hematopoietic cells with undifferentiated surface phenotype remains entirely within the CD34+CD38**−**CD90+ fraction of cultured cells.** Fractionation of CD34+CD38−CD90+ cells after 2 weeks of culture based on surface phenotype demonstrates sustained expansion of CD34+CD38−CD90+ cells during re-plating only within the CD34+CD38−CD90+ population. (A) Upper FACS plots represent the cells and gates used for the fractionation at 2 weeks and purity plots. (B) FACS plots in the middle show the immunophenotype of the fractions after additional 2 weeks of culture and the charts below summarize the total fold expansion of hematopoietic cells fractionated at (C) 2 weeks and (D) 5 weeks of culture. Error bars represent SEM (* p<0.05, n = 3).
(TIF)

Figure S5 **OP9M2 supports the expansion of cord blood (CB) CD34+CD38**−**CD90+ cells.** (A) Representative FACS plots of CD34+ CB cells after 2 and 8 weeks of co-culture on OP9M2 are shown. (B) Summary of total fold expansion of all CB cells from input cells cultured with or without OP9M2. (C) Summary of fold expansion of CD34+CD38−CD90+ CB cells cultured with or without OP9M2. Fractionation of cultured CB cells after 2 weeks of culture demonstrates sustained expansion of the undifferentiated CD34+CD38−CD90+ cells during re-plating only within the CD34+CD38−CD90+ population. (D) FACS plots represent the cells and gates used for the fractionation at 2 weeks. (E) FACS plots show the immunophenotype of the fractions after additional 2 weeks of culture and (F) summarize the total fold expansion of hematopoietic cells fractionated at 2 weeks and additional 2 weeks of culture (Error bars represent SEM (* p<0.05, n = 3).
(TIF)

Figure S6 **CD34+CD38**−**CD90+ cells expanded on OP9M2 demonstrate slight relative decrease in the frequency of hematopoietic progenitors.** (A) Frequency of CFUs among *ex vivo* expanded CD34+CD38−CD90+ cells. (B) Colony distribution among *ex vivo* expanded CD34+CD38−CD90+ cells. (C) Frequency of multipotent progenitors among *ex vivo* expanded CD34+CD38−CD90+ cells as measured by single cell assay. Error bars represent SEM (* p<0.05, ✦ p = 0.051, n = 3 independent experiments, except for C, only one experiment showed robust proliferation of single CD34+CD38−CD90+ cells sorted and re-plated at 5 weeks).
(TIF)

Figure S7 ***Ex vivo*** **expanded CD34+CD38**−**CD90+ cells demonstrate continued dependence on OP9M2 niche signals.** CD34+ FL cells cultured on OP9M2 for 2 weeks rapidly differentiate when transferred to non-supportive BFC012 stroma or no stroma. Left FACS plot: cells replated on OP9M2, Middle FACS plot: cells replated on BFC012, Right FACS plot: cells replated without stroma.
(TIF)

Figure S8 **CD34+CD38**−**CD90+ cells expanded on OP9M2 stroma undergo an initial culture shock resulting in transient activation of the aryl hydrocarbon receptor (AhR) signaling pathway.** Heat map showing genes in cultured fetal CD34+CD38−CD90+ cells, selected if they were at least two-fold downregulated in CD34+ cells treated with SR1 (inhibitor of AhR signaling) (GEO series GSE28359) [1], and at least two-fold up-regulated in cultured fetal liver CD34+CD38−CD90+ cells. All five arrays (untreated, and treated with four different concentrations of SR1) from Boitano et al [1] were normalized using RMA method and fold change was calculated comparing SR1 treated cells to the untreated control. Thirteen genes were at least 2-fold down-regulated in two or more concentrations of SR1 treated cells. Of those, 9 were differentially expressed in our dataset of cultured CD34+CD38−CD90+ cells (illustrated in heat map above). The heat map was generated on probe sets called as “Present” using the MAS5 algorithm in at least in one sample and if the absolute expression level was above 50. Several of these genes are known members of the AHR signaling pathway, which is a negative regulator of HSC function [1], [2]. Robust activation of this pathway was observed in CD34+CD38−CD90+ cells cultured for 12 hours, with restoration to almost normal levels by 2 weeks. AHRR negatively regulates AHR, however, it’s own transcription is regulated by AHR activation [3], [4]. CYP1B1 is also regulated by AHR activation [1]. Interestingly, the inflammatory cytokine, CCL2, has been linked to activation of mTOR [5], which compromises HSC function. Therefore, activation of AHR signaling and inflammatory pathways at the onset of *ex vivo* culture may trigger downstream effectors that negatively regulate CD34+CD38−CD90+ cell function.
(TIF)

Table S1 Reconstitution and lineage distribution of human hematopoietic cells in transplanted NSG mice. The first column indicates the number of input CD34+ cells (before culture) each mouse was transplanted with. %hCD45 indicates the percentage of total human hematopoietic engraftment. Lineage distribution is shown as percentage of total human CD45 engraftment for each organ.
(XLS)

Table S2 Expression of known HSC regulators in ex vivo expanded CD34+CD38−CD90+cells. Genes are divided by their cellular location defined by IPA. MeanExp represents mean gene expression values of the replicates. Fold change and p-value for differential expression between freshly isolated (day 0) and cultured (12 h, 2 weeks and 5 weeks) CD34+CD38−CD90+cells were calculated from the M-value reported by Limma. PMA indicates absent (A), marginal (M) and present (P) calls for each replicate. GO location and GO function for each gene is defined by DAVID, IPA gene location and type of gene are defined by IPA. Ref represents publications documenting the role of each gene in HSC development or maintenance. Bold numbers indicate probes that were significantly changed (>2 fold, p<0.05) in comparison to the expression value at day 0. Probes in bold are significantly differentially expressed.
(XLS)

Table S3 Cluster H. Gene expression changes of *ex vivo* expanded CD34+CD38−CD90+cells. Each worksheet contains a specific fuzzy-c means cluster, as represented in Figure 5. Genes are divided by their cellular location defined by Ingenuity Pathway Analysis (IPA). MeanExp represents mean gene expression values of the replicates (Day 0, 12 hr and 2 weeks contain 3 replicates; 5 weeks contains 2 replicates). Fold change and p-value for differential expression between Day 0 and cultured cells are from Limma. PMA indicates absent (A), marginal (M) and present (P) calls for each replicate. GO location and GO function for each gene is defined by DAVID, IPA gene location and type of gene are defined by IPA.
(ZIP)

References S1 (TIF)

## References

[1] Bordignon C (2006) Stem-cell therapies for blood diseases. Nature 441: 1100–1102. 16810247 10.1038/nature04962

[2] Weissman IL (2000) Stem cells: units of development, units of regeneration, and units in evolution. Cell 100: 157–168. 10647940 10.1016/s0092-8674(00)81692-x

[3] Stanevsky A, Goldstein G, Nagler A (2009) Umbilical cord blood transplantation: pros, cons and beyond. Blood Rev 23: 199–204. 19282073 10.1016/j.blre.2009.02.001

[4] Teitell MA, Mikkola HK (2006) Transcriptional activators, repressors, and epigenetic modifiers controlling hematopoietic stem cell development. Pediatr Res 59: 33R–39R. 16549546 10.1203/01.pdr.0000205155.26315.c7

[5] Nakauchi H, Sudo K, Ema H (2001) Quantitative assessment of the stem cell self-renewal capacity. Ann N Y Acad Sci 938: 18–24; discussion 24–15. 10.1111/j.1749-6632.2001.tb03570.x 11458506

[6] Naveiras O, Nardi V, Wenzel PL, Hauschka PV, Fahey F, et al (2009) Bone-marrow adipocytes as negative regulators of the haematopoietic microenvironment. Nature 460: 259–263. 19516257 10.1038/nature08099 PMC2831539

[7] Chow A, Lucas D, Hidalgo A, Mendez-Ferrer S, Hashimoto D, et al (2011) Bone marrow CD169+ macrophages promote the retention of hematopoietic stem and progenitor cells in the mesenchymal stem cell niche. J Exp Med 208: 261–271. 21282381 10.1084/jem.20101688 PMC3039855

[8] Calvi LM, Adams GB, Weibrecht KW, Weber JM, Olson DP, et al (2003) Osteoblastic cells regulate the haematopoietic stem cell niche. Nature 425: 841–846. 14574413 10.1038/nature02040

[9] Mendez-Ferrer S, Michurina TV, Ferraro F, Mazloom AR, Macarthur BD, et al (2010) Mesenchymal and haematopoietic stem cells form a unique bone marrow niche. Nature 466: 829–834. 20703299 10.1038/nature09262 PMC3146551

[10] Kobayashi H, Butler JM, O’Donnell R, Kobayashi M, Ding BS, et al (2010) Angiocrine factors from Akt-activated endothelial cells balance self-renewal and differentiation of haematopoietic stem cells. Nat Cell Biol 12: 1046–1056. 20972423 10.1038/ncb2108 PMC2972406

[11] Arai F, Hirao A, Ohmura M, Sato H, Matsuoka S, et al (2004) Tie2/angiopoietin-1 signaling regulates hematopoietic stem cell quiescence in the bone marrow niche. Cell 118: 149–161. 15260986 10.1016/j.cell.2004.07.004

[12] Zhang J, Niu C, Ye L, Huang H, He X, et al (2003) Identification of the haematopoietic stem cell niche and control of the niche size. Nature 425: 836–841. 14574412 10.1038/nature02041

[13] Bryder D, Jacobsen SE (2000) Interleukin-3 supports expansion of long-term multilineage repopulating activity after multiple stem cell divisions in vitro. Blood 96: 1748–1755. 10961873

[14] Zhang CC, Kaba M, Ge G, Xie K, Tong W, et al (2006) Angiopoietin-like proteins stimulate ex vivo expansion of hematopoietic stem cells. Nat Med 12: 240–245. 16429146 10.1038/nm1342 PMC2771412

[15] Miller CL, Eaves CJ (1997) Expansion in vitro of adult murine hematopoietic stem cells with transplantable lympho-myeloid reconstituting ability. Proc Natl Acad Sci U S A 94: 13648–13653. 9391080 10.1073/pnas.94.25.13648 PMC28360

[16] Delaney C, Heimfeld S, Brashem-Stein C, Voorhies H, Manger RL, et al (2010) Notch-mediated expansion of human cord blood progenitor cells capable of rapid myeloid reconstitution. Nat Med 16: 232–236. 20081862 10.1038/nm.2080 PMC2819359

[17] Antonchuk J, Sauvageau G, Humphries RK (2002) HOXB4-induced expansion of adult hematopoietic stem cells ex vivo. Cell 109: 39–45. 11955445 10.1016/s0092-8674(02)00697-9

[18] Amsellem S, Pflumio F, Bardinet D, Izac B, Charneau P, et al (2003) Ex vivo expansion of human hematopoietic stem cells by direct delivery of the HOXB4 homeoprotein. Nat Med 9: 1423–1427. 14578882 10.1038/nm953

[19] Boitano AE, Wang J, Romeo R, Bouchez LC, Parker AE, et al (2010) Aryl hydrocarbon receptor antagonists promote the expansion of human hematopoietic stem cells. Science 329: 1345–1348. 20688981 10.1126/science.1191536 PMC3033342

[20] Oostendorp RA, Harvey KN, Kusadasi N, de Bruijn MF, Saris C, et al (2002) Stromal cell lines from mouse aorta-gonads-mesonephros subregions are potent supporters of hematopoietic stem cell activity. Blood 99: 1183–1189. 11830464 10.1182/blood.v99.4.1183

[21] Moore KA, Ema H, Lemischka IR (1997) In vitro maintenance of highly purified, transplantable hematopoietic stem cells. Blood 89: 4337–4347. 9192756

[22] Shimakura Y, Kawada H, Ando K, Sato T, Nakamura Y, et al (2000) Murine stromal cell line HESS-5 maintains reconstituting ability of Ex vivo-generated hematopoietic stem cells from human bone marrow and cytokine-mobilized peripheral blood. Stem Cells 18: 183–189. 10840071 10.1634/stemcells.18-3-183

[23] Nolta JA, Thiemann FT, Arakawa-Hoyt J, Dao MA, Barsky LW, et al (2002) The AFT024 stromal cell line supports long-term ex vivo maintenance of engrafting multipotent human hematopoietic progenitors. Leukemia 16: 352–361. 11896538 10.1038/sj.leu.2402371

[24] Vanheusden K, Van Coppernolle S, De Smedt M, Plum J, Vandekerckhove B (2007) In vitro expanded cells contributing to rapid severe combined immunodeficient repopulation activity are CD34+38–33+90+45RA. Stem Cells 25: 107–114. 16973833 10.1634/stemcells.2006-0256

[25] Sutherland HJ, Eaves CJ, Eaves AC, Dragowska W, Lansdorp PM (1989) Characterization and partial purification of human marrow cells capable of initiating long-term hematopoiesis in vitro. Blood 74: 1563–1570. 2790186

[26] Nakano T, Kodama H, Honjo T (1994) Generation of lymphohematopoietic cells from embryonic stem cells in culture. Science 265: 1098–1101. 8066449 10.1126/science.8066449

[27] Hackney JA, Charbord P, Brunk BP, Stoeckert CJ, Lemischka IR, et al (2002) A molecular profile of a hematopoietic stem cell niche. Proc Natl Acad Sci U S A 99: 13061–13066. 12226475 10.1073/pnas.192124499 PMC130586

[28] Collins LS, Dorshkind K (1987) A stromal cell line from myeloid long-term bone marrow cultures can support myelopoiesis and B lymphopoiesis. J Immunol 138: 1082–1087. 3492541

[29] Itoh K, Tezuka H, Sakoda H, Konno M, Nagata K, et al (1989) Reproducible establishment of hemopoietic supportive stromal cell lines from murine bone marrow. Exp Hematol 17: 145–153. 2783573

[30] Gentleman RC, Carey VJ, Bates DM, Bolstad B, Dettling M, et al (2004) Bioconductor: open software development for computational biology and bioinformatics. Genome Biol 5: R80. 15461798 10.1186/gb-2004-5-10-r80 PMC545600

[31] Wettenhall JM, Simpson KM, Satterley K, Smyth GK (2006) affylmGUI: a graphical user interface for linear modeling of single channel microarray data. Bioinformatics 22: 897–899. 16455752 10.1093/bioinformatics/btl025

[32] Gao J, Yan XL, Li R, Liu Y, He W, et al (2010) Characterization of OP9 as authentic mesenchymal stem cell line. J Genet Genomics 37: 475–482. 20659712 10.1016/S1673-8527(09)60067-9

[33] Majeti R, Park CY, Weissman IL (2007) Identification of a hierarchy of multipotent hematopoietic progenitors in human cord blood. Cell Stem Cell 1: 635–645. 18371405 10.1016/j.stem.2007.10.001 PMC2292126

[34] Dorrell C, Gan OI, Pereira DS, Hawley RG, Dick JE (2000) Expansion of human cord blood CD34(+)CD38(−) cells in ex vivo culture during retroviral transduction without a corresponding increase in SCID repopulating cell (SRC) frequency: dissociation of SRC phenotype and function. Blood 95: 102–110. 10607692

[35] Nutt SL, Heavey B, Rolink AG, Busslinger M (1999) Commitment to the B-lymphoid lineage depends on the transcription factor Pax5. Nature 401: 556–562. 10524622 10.1038/44076

[36] Iwasaki H, Somoza C, Shigematsu H, Duprez EA, Iwasaki-Arai J, et al (2005) Distinctive and indispensable roles of PU.1 in maintenance of hematopoietic stem cells and their differentiation. Blood 106: 1590–1600. 15914556 10.1182/blood-2005-03-0860 PMC1895212

[37] Pevny L, Simon MC, Robertson E, Klein WH, Tsai SF, et al (1991) Erythroid differentiation in chimaeric mice blocked by a targeted mutation in the gene for transcription factor GATA-1. Nature 349: 257–260. 1987478 10.1038/349257a0

[38] Zhang P, Iwasaki-Arai J, Iwasaki H, Fenyus ML, Dayaram T, et al (2004) Enhancement of hematopoietic stem cell repopulating capacity and self-renewal in the absence of the transcription factor C/EBP alpha. Immunity 21: 853–863. 15589173 10.1016/j.immuni.2004.11.006

[39] Liu ZG (2005) Molecular mechanism of TNF signaling and beyond. Cell Res 15: 24–27. 15686622 10.1038/sj.cr.7290259

[40] Dohn M, Zhang S, Chen X (2001) p63alpha and DeltaNp63alpha can induce cell cycle arrest and apoptosis and differentially regulate p53 target genes. Oncogene 20: 3193–3205. 11423969 10.1038/sj.onc.1204427

[41] Cheng T, Rodrigues N, Shen H, Yang Y, Dombkowski D, et al (2000) Hematopoietic stem cell quiescence maintained by p21cip1/waf1. Science 287: 1804–1808. 10710306 10.1126/science.287.5459.1804

[42] Shojaei F, Trowbridge J, Gallacher L, Yuefei L, Goodale D, et al (2005) Hierarchical and ontogenic positions serve to define the molecular basis of human hematopoietic stem cell behavior. Dev Cell 8: 651–663. 15866157 10.1016/j.devcel.2005.03.004

[43] Mackarehtschian K, Hardin JD, Moore KA, Boast S, Goff SP, et al (1995) Targeted disruption of the flk2/flt3 gene leads to deficiencies in primitive hematopoietic progenitors. Immunity 3: 147–161. 7621074 10.1016/1074-7613(95)90167-1

[44] Matsumoto A, Takeishi S, Kanie T, Susaki E, Onoyama I, et al (2011) p57 is required for quiescence and maintenance of adult hematopoietic stem cells. Cell Stem Cell 9: 262–271. 21885021 10.1016/j.stem.2011.06.014

[45] Suda T, Takubo K, Semenza GL (2011) Metabolic regulation of hematopoietic stem cells in the hypoxic niche. Cell Stem Cell 9: 298–310. 21982230 10.1016/j.stem.2011.09.010

[46] Carvajal LA, Hamard PJ, Tonnessen C, Manfredi JJ (2012) E2F7, a novel target, is up-regulated by p53 and mediates DNA damage-dependent transcriptional repression. Genes Dev 26: 1533–1545. 22802528 10.1101/gad.184911.111 PMC3404382

[47] Radomska HS, Huettner CS, Zhang P, Cheng T, Scadden DT, et al (1998) CCAAT/enhancer binding protein alpha is a regulatory switch sufficient for induction of granulocytic development from bipotential myeloid progenitors. Mol Cell Biol 18: 4301–4314. 9632814 10.1128/mcb.18.7.4301 PMC109014

[48] Austin GE, Chan WC, Zhao W, Racine M (1994) Myeloperoxidase gene expression in normal granulopoiesis and acute leukemias. Leuk Lymphoma 15: 209–226. 7866270 10.3109/10428199409049717

[49] Elsbach P (1998) The bactericidal/permeability-increasing protein (BPI) in antibacterial host defense. J Leukoc Biol 64: 14–18. 9665269 10.1002/jlb.64.1.14

[50] Sudo T, Nishikawa S, Ohno N, Akiyama N, Tamakoshi M, et al (1993) Expression and function of the interleukin 7 receptor in murine lymphocytes. Proc Natl Acad Sci U S A 90: 9125–9129. 8415665 10.1073/pnas.90.19.9125 PMC47514

[51] Oettinger MA, Schatz DG, Gorka C, Baltimore D (1990) RAG-1 and RAG-2, adjacent genes that synergistically activate V(D)J recombination. Science 248: 1517–1523. 2360047 10.1126/science.2360047

[52] Ball LM, Bernardo ME, Roelofs H, Lankester A, Cometa A, et al (2007) Cotransplantation of ex vivo expanded mesenchymal stem cells accelerates lymphocyte recovery and may reduce the risk of graft failure in haploidentical hematopoietic stem-cell transplantation. Blood 110: 2764–2767. 17638847 10.1182/blood-2007-04-087056

[53] Le Blanc K, Ringden O (2007) Immunomodulation by mesenchymal stem cells and clinical experience. J Intern Med 262: 509–525. 17949362 10.1111/j.1365-2796.2007.01844.x

[54] Ficara F, Murphy MJ, Lin M, Cleary ML (2008) Pbx1 regulates self-renewal of long-term hematopoietic stem cells by maintaining their quiescence. Cell Stem Cell 2: 484–496. 18462698 10.1016/j.stem.2008.03.004 PMC2416441

[55] Min IM, Pietramaggiori G, Kim FS, Passegue E, Stevenson KE, et al (2008) The transcription factor EGR1 controls both the proliferation and localization of hematopoietic stem cells. Cell Stem Cell 2: 380–391. 18397757 10.1016/j.stem.2008.01.015

[56] Ku CJ, Hosoya T, Maillard I, Engel JD (2012) GATA-3 regulates hematopoietic stem cell maintenance and cell-cycle entry. Blood 119: 2242–2251. 22267605 10.1182/blood-2011-07-366070 PMC3311255

[57] Buza-Vidas N, Duarte S, Luc S, Bouriez-Jones T, Woll PS, et al (2011) GATA3 is redundant for maintenance and self-renewal of hematopoietic stem cells. Blood 118: 1291–1293. 21670475 10.1182/blood-2011-02-338046

[58] Mao Y, Tournier AL, Bates PA, Gale JE, Tapon N, et al (2011) Planar polarization of the atypical myosin Dachs orients cell divisions in Drosophila. Genes Dev 25: 131–136. 21245166 10.1101/gad.610511 PMC3022259

[59] Bultje RS, Castaneda-Castellanos DR, Jan LY, Jan YN, Kriegstein AR, et al (2009) Mammalian Par3 regulates progenitor cell asymmetric division via notch signaling in the developing neocortex. Neuron 63: 189–202. 19640478 10.1016/j.neuron.2009.07.004 PMC2736606

[60] Fukuda MN, Sato T, Nakayama J, Klier G, Mikami M, et al (1995) Trophinin and tastin, a novel cell adhesion molecule complex with potential involvement in embryo implantation. Genes Dev 9: 1199–1210. 7758945 10.1101/gad.9.10.1199

[61] Pina C, May G, Soneji S, Hong D, Enver T (2008) MLLT3 regulates early human erythroid and megakaryocytic cell fate. Cell Stem Cell 2: 264–273. 18371451 10.1016/j.stem.2008.01.013

[62] Challen GA, Sun D, Jeong M, Luo M, Jelinek J, et al (2012) Dnmt3a is essential for hematopoietic stem cell differentiation. Nat Genet 44: 23–31. 10.1038/ng.1009 PMC3637952 22138693

[63] Hope KJ, Cellot S, Ting SB, MacRae T, Mayotte N, et al (2010) An RNAi screen identifies Msi2 and Prox1 as having opposite roles in the regulation of hematopoietic stem cell activity. Cell Stem Cell 7: 101–113. 20621054 10.1016/j.stem.2010.06.007

[64] Cai M, Langer EM, Gill JG, Satpathy AT, Albring JC, et al (2012) Dual actions of Meis1 inhibit erythroid progenitor development and sustain general hematopoietic cell proliferation. Blood 120: 335–346. 22665933 10.1182/blood-2012-01-403139 PMC3628121

[65] Holyoake TL, Nicolini FE, Eaves CJ (1999) Functional differences between transplantable human hematopoietic stem cells from fetal liver, cord blood, and adult marrow. Exp Hematol 27: 1418–1427. 10480433 10.1016/s0301-472x(99)00078-8

[66] Krosl J, Beslu N, Mayotte N, Humphries RK, Sauvageau G (2003) The competitive nature of HOXB4-transduced HSC is limited by PBX1: the generation of ultra-competitive stem cells retaining full differentiation potential. Immunity 18: 561–571. 12705858 10.1016/s1074-7613(03)00090-6

[67] Ivanovic Z, Dello Sbarba P, Trimoreau F, Faucher JL, Praloran V (2000) Primitive human HPCs are better maintained and expanded in vitro at 1 percent oxygen than at 20 percent. Transfusion 40: 1482–1488. 11134568 10.1046/j.1537-2995.2000.40121482.x

[68] Danet GH, Pan Y, Luongo JL, Bonnet DA, Simon MC (2003) Expansion of human SCID-repopulating cells under hypoxic conditions. J Clin Invest 112: 126–135. 12840067 10.1172/JCI17669 PMC162287

[69] Jude CD, Gaudet JJ, Speck NA, Ernst P (2008) Leukemia and hematopoietic stem cells: balancing proliferation and quiescence. Cell Cycle 7: 586–591. 18239455 10.4161/cc.7.5.5549 PMC2892629

