# Supplementary figures and images for: Expansion on Stromal Cells Preserves the Undifferentiated State of Human Hematopoietic Stem Cells Despite Compromised Reconstitution Ability
Source: PLoS One. 2013 Jan 16;8(1):e53912. doi: 10.1371/journal.pone.0053912 (PMC3547050; doi:10.1371/journal.pone.0053912)

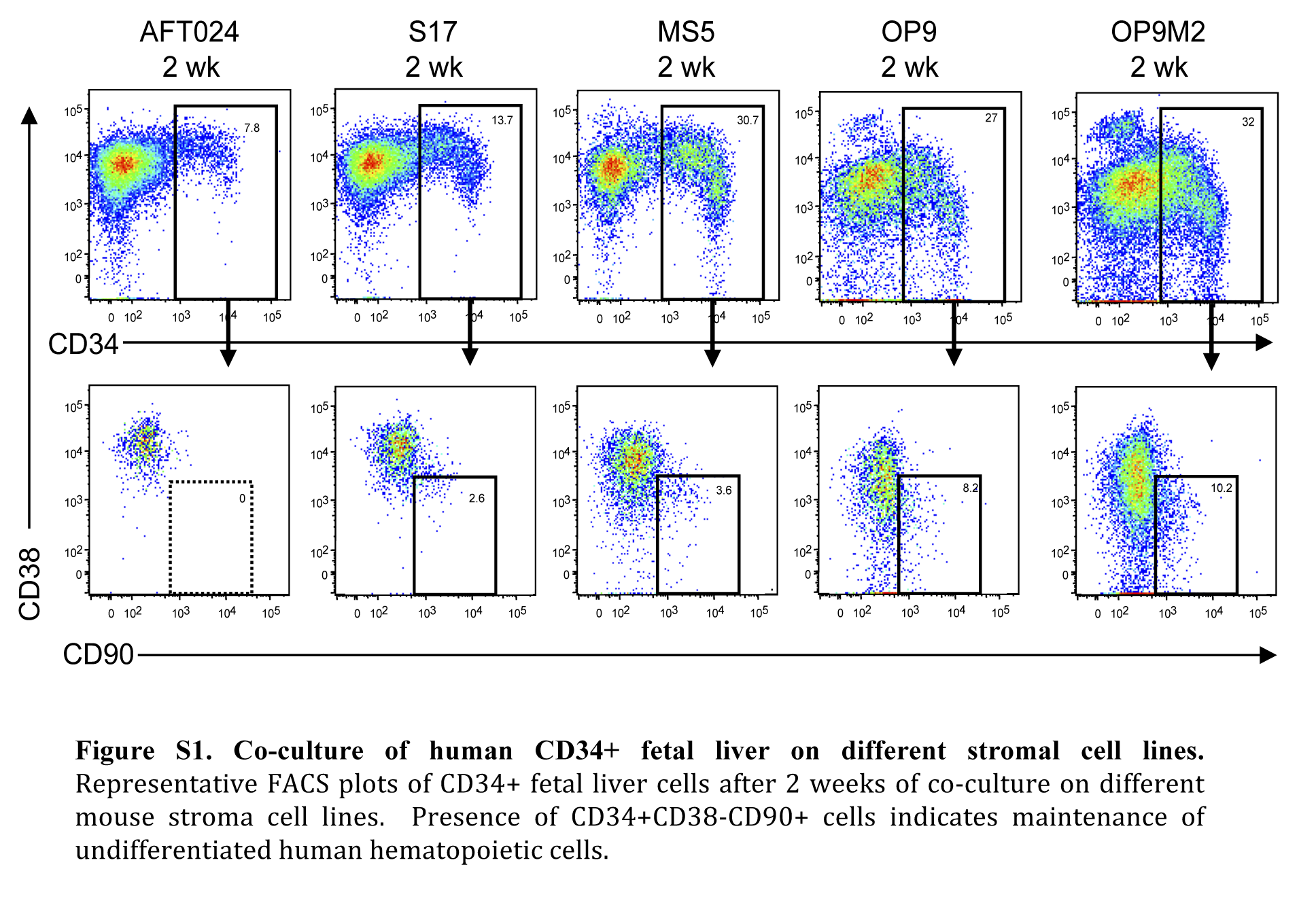

Supplement: Figure S1 — Co-culture of human CD34+ fetal liver on different stromal cell lines. Representative FACS plots of CD34+ fetal liver cells after 2 weeks of co-culture on different mouse stroma cell lines. Presence of CD34+CD38−CD90+ cells indicates maintenance of undifferentiated human hematopoietic cells. (TIF) [file pone.0053912.s001.tif]

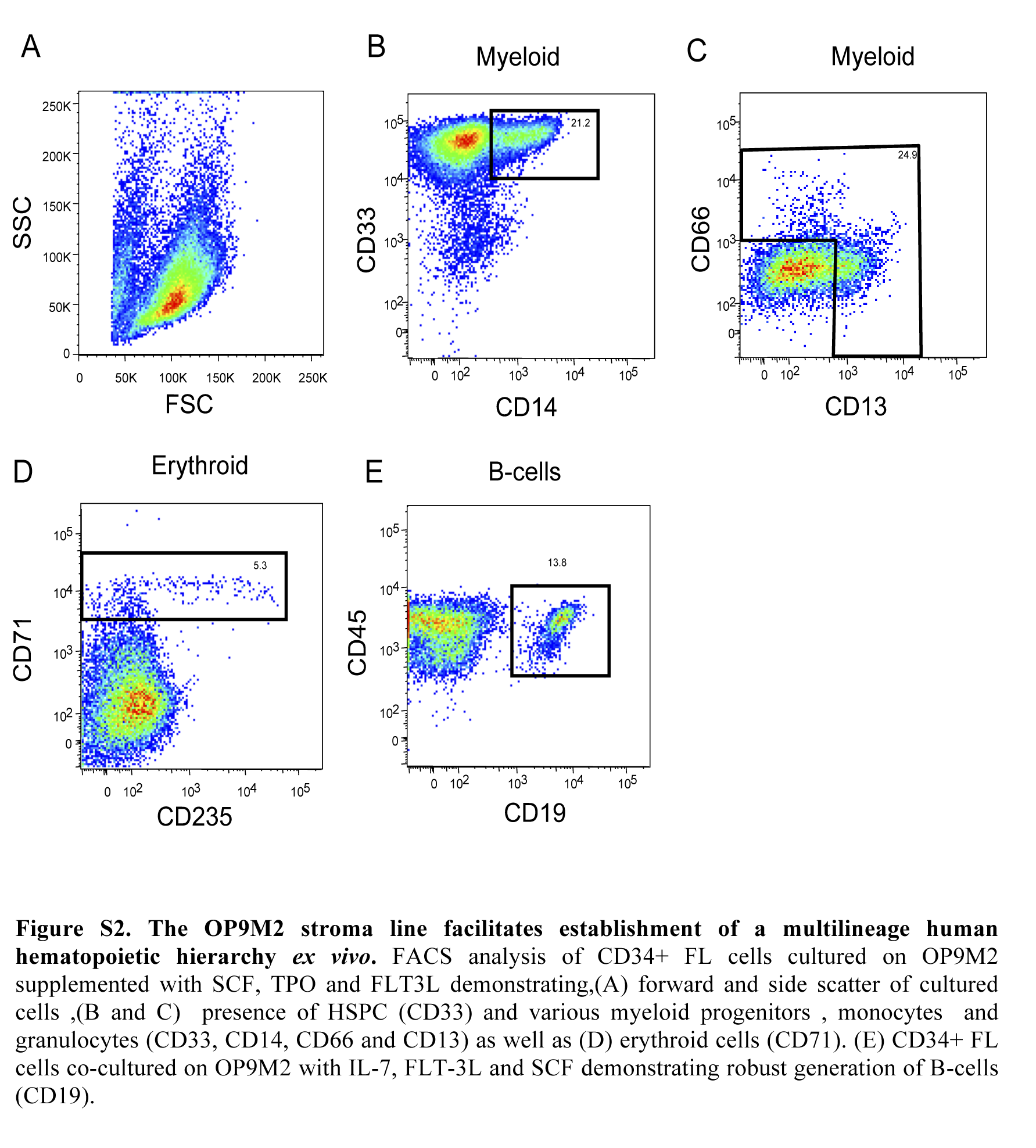

Supplement: Figure S2 — The OP9M2 stroma line facilitates establishment of a multilineage human hematopoietic hierarchy ex vivo . FACS analysis of CD34+ FL cells cultured on OP9M2 supplemented with SCF, TPO and FLT3L demonstrating, (A) forward and side scatter of cultured cells, (B and C) presence of HSPC (CD33) and various myeloid progenitors, monocytes and granulocytes (CD33, CD14, CD66 and CD13) as well as (D) erythroid cells (CD71). (E) CD34+ FL cells co-cultured on OP9M2 with IL-7, FLT-3L and SCF demonstrating robust generation of B-cells (CD19). (TIF) [file pone.0053912.s002.tif]

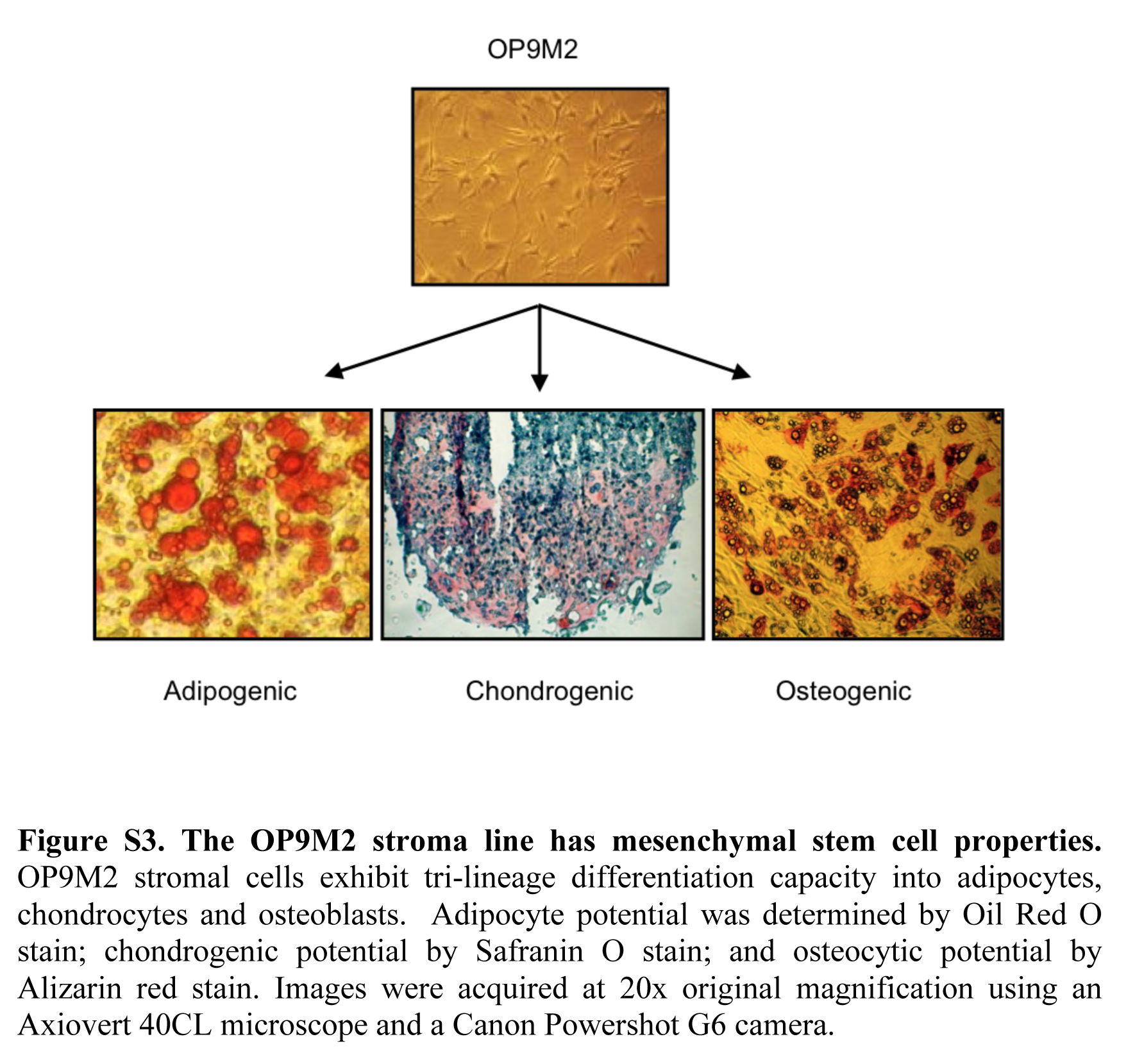

Supplement: Figure S3 — The OP9M2 stroma line has mesenchymal stem cell properties. OP9M2 stromal cells exhibit tri-lineage differentiation capacity into adipocytes, chondrocytes and osteoblasts. Adipocyte potential was determined by Oil Red O stain; chondrogenic potential by Safranin O stain; and osteocytic potential by Alizarin red stain. Images were acquired at 20× original magnification using an Axiovert 40CL microscope and a Canon Powershot G6 camera. (TIF) [file pone.0053912.s003.tif]

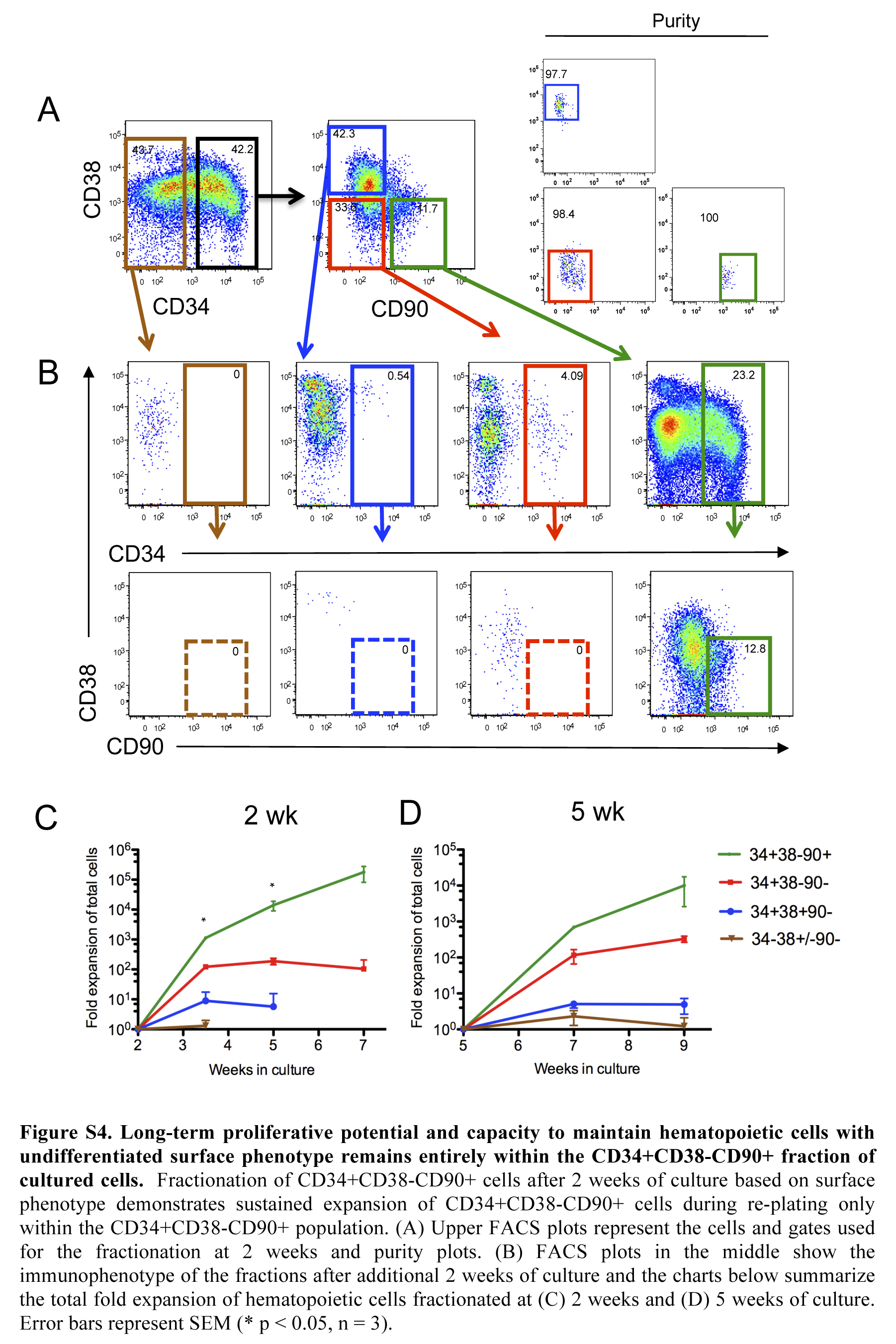

Supplement: Figure S4 — Long-term proliferative potential and capacity to maintain hematopoietic cells with undifferentiated surface phenotype remains entirely within the CD34+CD38−CD90+ fraction of cultured cells. Fractionation of CD34+CD38−CD90+ cells after 2 weeks of culture based on surface phenotype demonstrates sustained expansion of CD34+CD38−CD90+ cells during re-plating only within the CD34+CD38−CD90+ population. (A) Upper FACS plots represent the cells and gates used for the fractionation at 2 weeks and purity plots. (B) FACS plots in the middle show the immunophenotype of the fractions after additional 2 weeks of culture and the charts below summarize the total fold expansion of hematopoietic cells fractionated at (C) 2 weeks and (D) 5 weeks of culture. Error bars represent SEM (* p<0.05, n = 3). (TIF) [file pone.0053912.s004.tif]

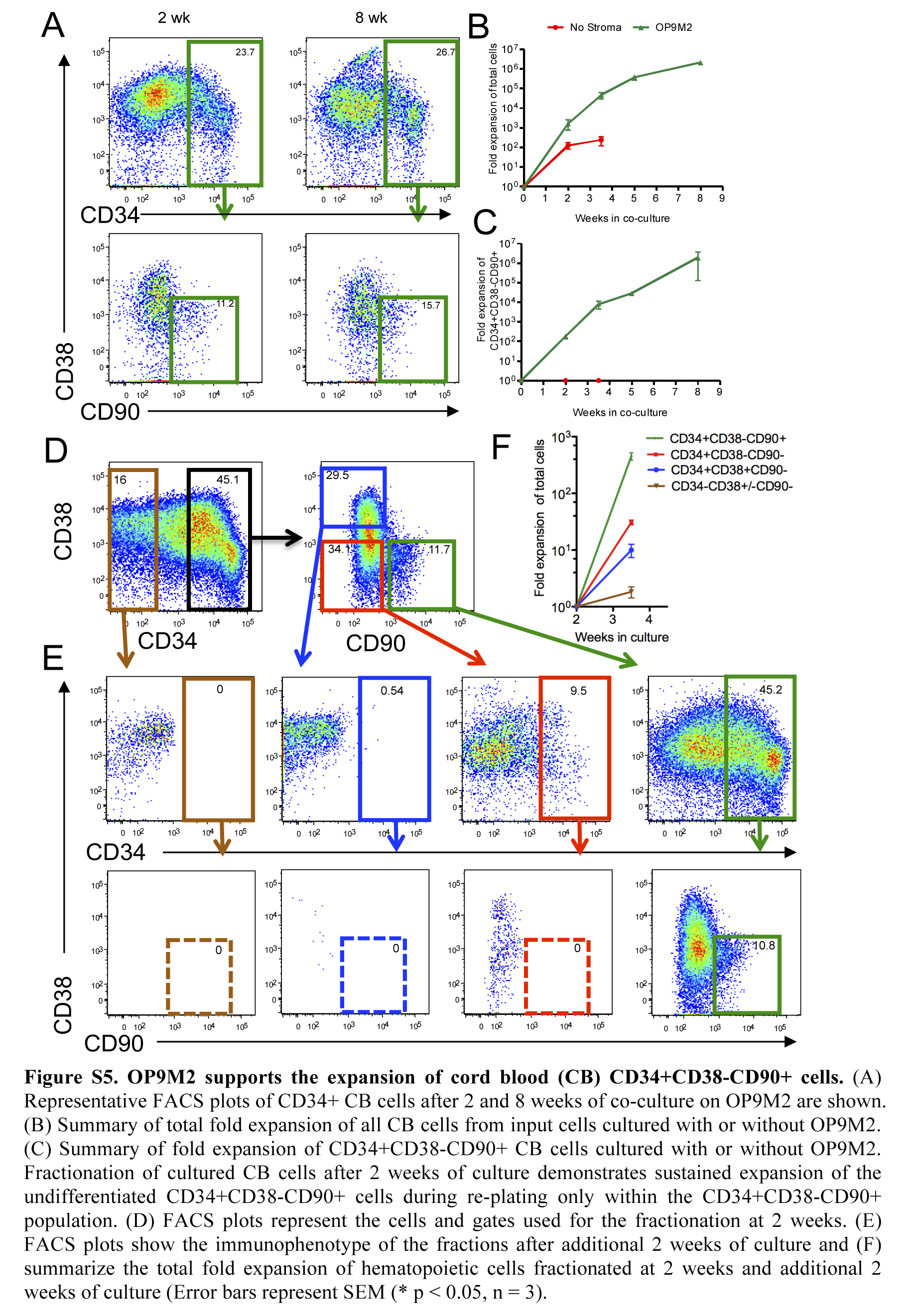

Supplement: Figure S5 — OP9M2 supports the expansion of cord blood (CB) CD34+CD38−CD90+ cells. (A) Representative FACS plots of CD34+ CB cells after 2 and 8 weeks of co-culture on OP9M2 are shown. (B) Summary of total fold expansion of all CB cells from input cells cultured with or without OP9M2. (C) Summary of fold expansion of CD34+CD38−CD90+ CB cells cultured with or without OP9M2. Fractionation of cultured CB cells after 2 weeks of culture demonstrates sustained expansion of the undifferentiated CD34+CD38−CD90+ cells during re-plating only within the CD34+CD38−CD90+ population. (D) FACS plots represent the cells and gates used for the fractionation at 2 weeks. (E) FACS plots show the immunophenotype of the fractions after additional 2 weeks of culture and (F) summarize the total fold expansion of hematopoietic cells fractionated at 2 weeks and additional 2 weeks of culture (Error bars represent SEM (* p<0.05, n = 3). (TIF) [file pone.0053912.s005.tif]

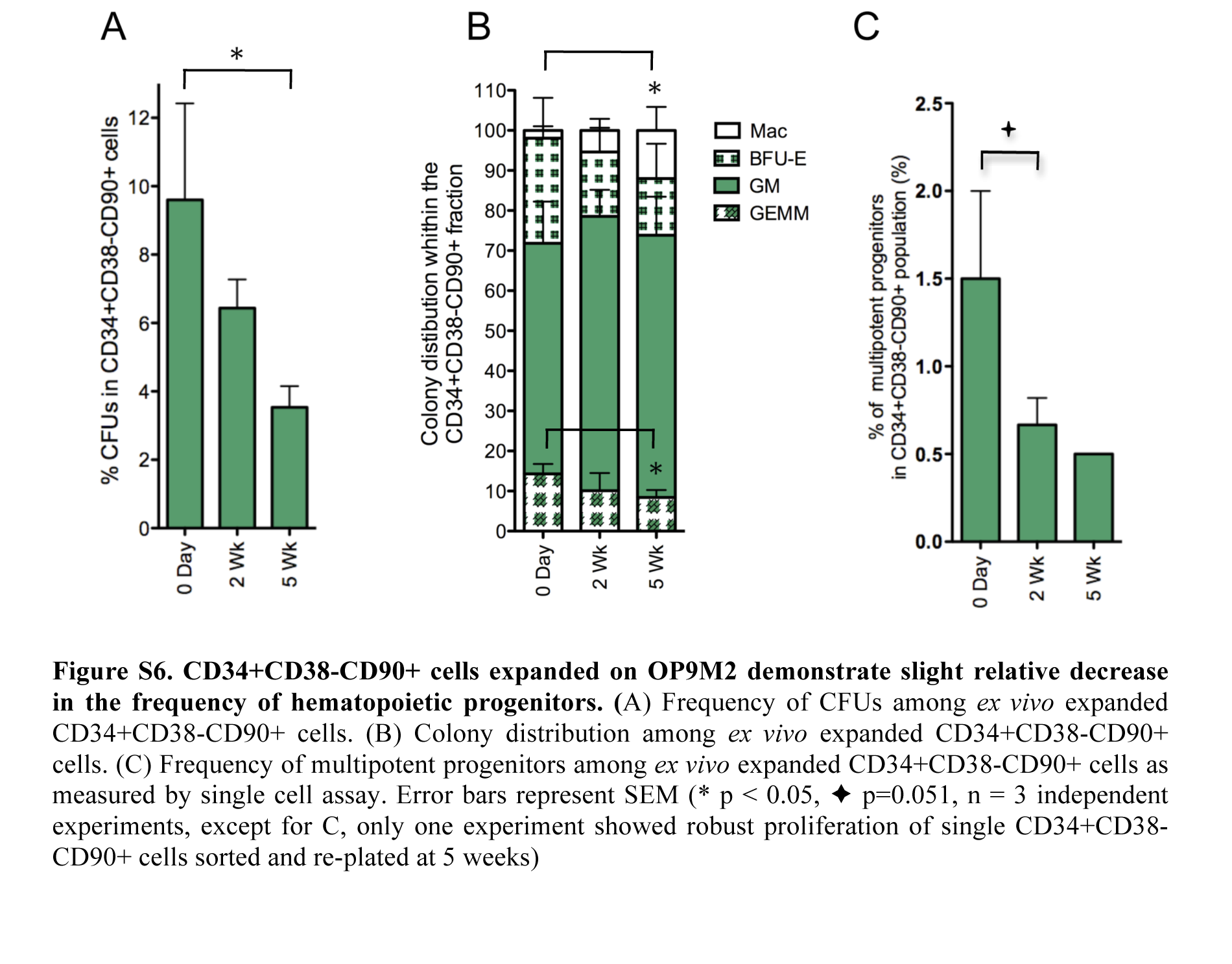

Supplement: Figure S6 — CD34+CD38−CD90+ cells expanded on OP9M2 demonstrate slight relative decrease in the frequency of hematopoietic progenitors. (A) Frequency of CFUs among ex vivo expanded CD34+CD38−CD90+ cells. (B) Colony distribution among ex vivo expanded CD34+CD38−CD90+ cells. (C) Frequency of multipotent progenitors among ex vivo expanded CD34+CD38−CD90+ cells as measured by single cell assay. Error bars represent SEM (* p<0.05, ✦ p = 0.051, n = 3 independent experiments, except for C, only one experiment showed robust proliferation of single CD34+CD38−CD90+ cells sorted and re-plated at 5 weeks). (TIF) [file pone.0053912.s006.tif]

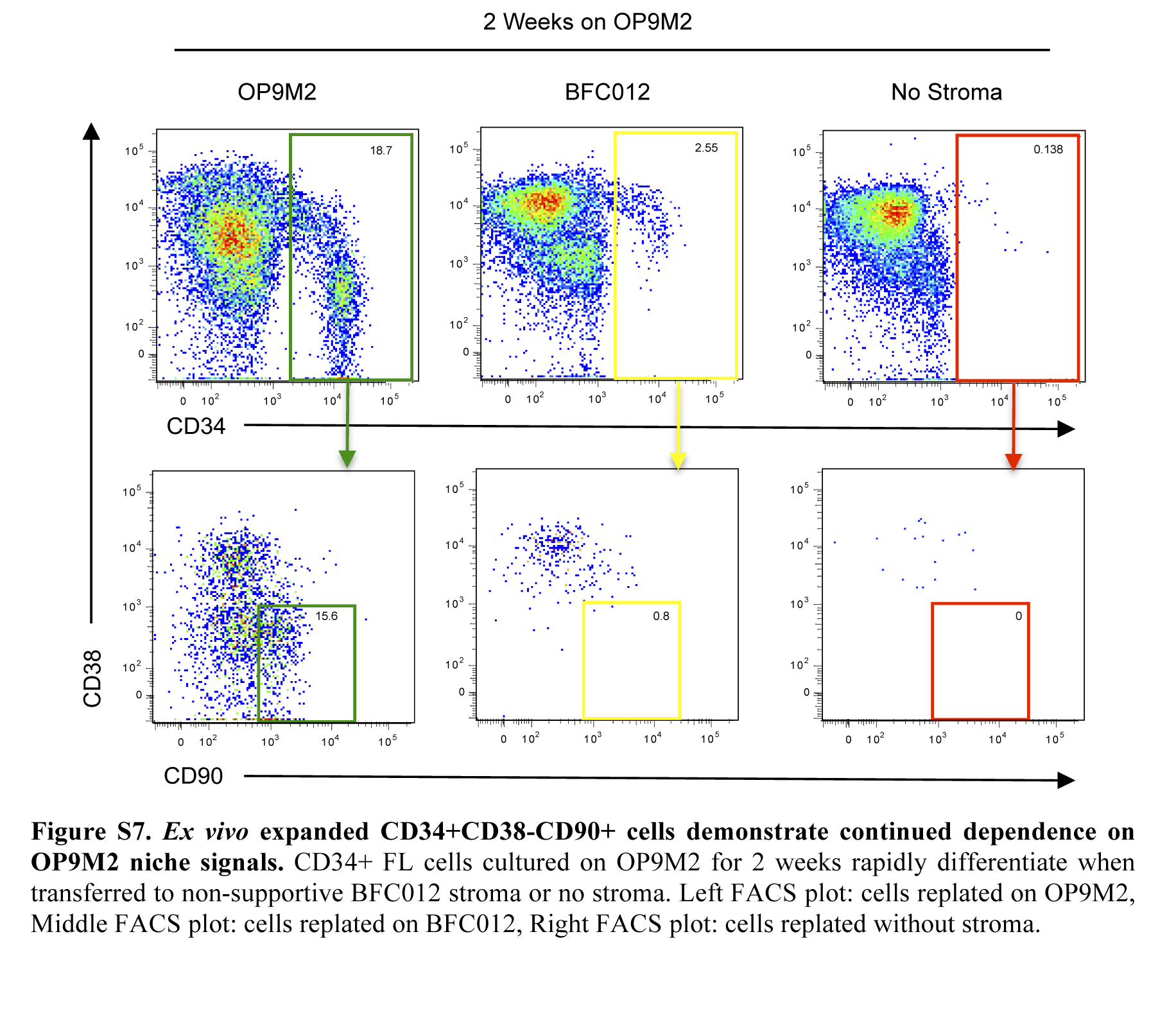

Supplement: Figure S7 — Ex vivo expanded CD34+CD38−CD90+ cells demonstrate continued dependence on OP9M2 niche signals. CD34+ FL cells cultured on OP9M2 for 2 weeks rapidly differentiate when transferred to non-supportive BFC012 stroma or no stroma. Left FACS plot: cells replated on OP9M2, Middle FACS plot: cells replated on BFC012, Right FACS plot: cells replated without stroma. (TIF) [file pone.0053912.s007.tif]

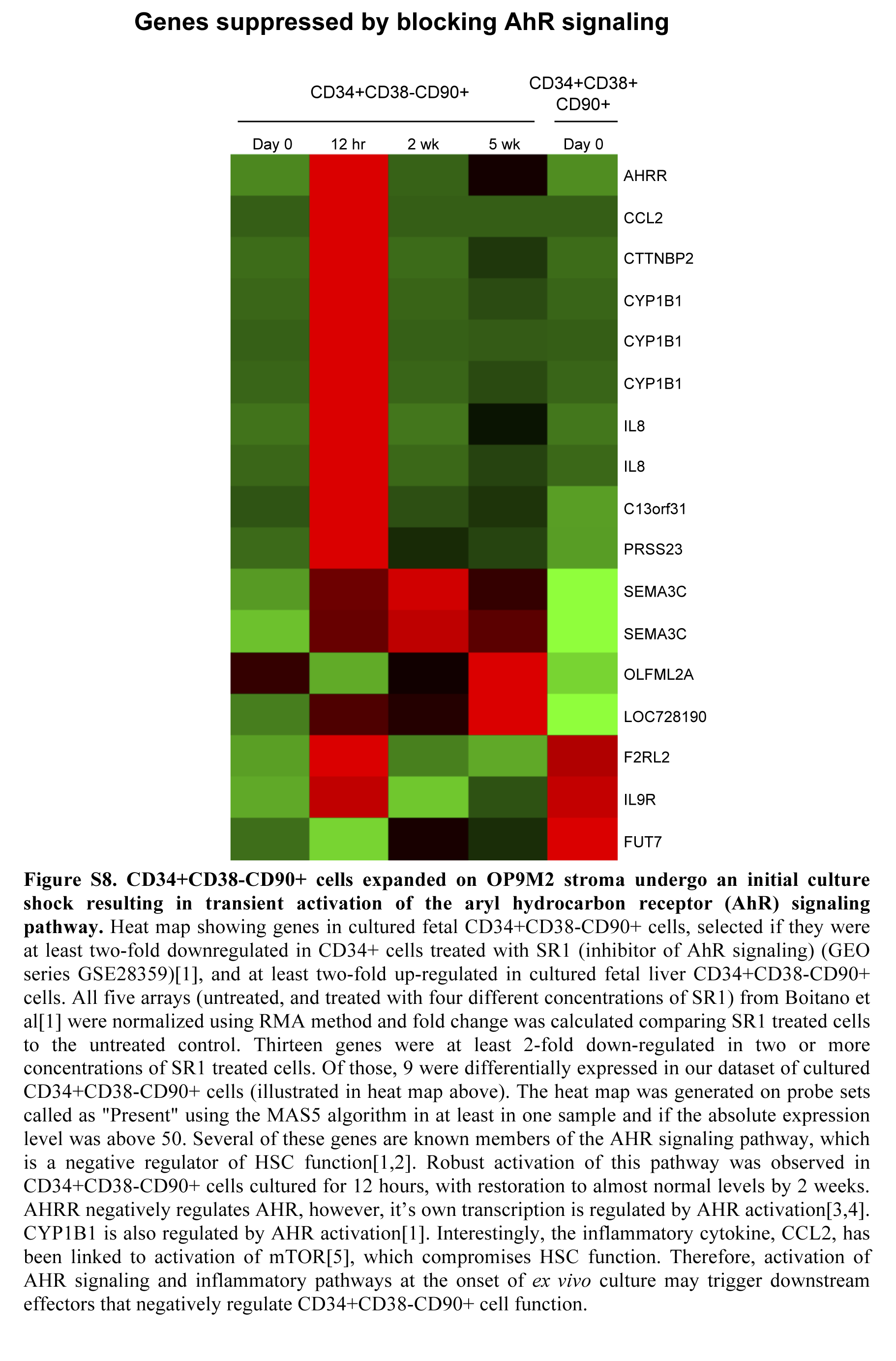

Supplement: Figure S8 — CD34+CD38−CD90+ cells expanded on OP9M2 stroma undergo an initial culture shock resulting in transient activation of the aryl hydrocarbon receptor (AhR) signaling pathway. Heat map showing genes in cultured fetal CD34+CD38−CD90+ cells, selected if they were at least two-fold downregulated in CD34+ cells treated with SR1 (inhibitor of AhR signaling) (GEO series GSE28359) [1], and at least two-fold up-regulated in cultured fetal liver CD34+CD38−CD90+ cells. All five arrays (untreated, and treated with four different concentrations of SR1) from Boitano et al [1] were normalized using RMA method and fold change was calculated comparing SR1 treated cells to the untreated control. Thirteen genes were at least 2-fold down-regulated in two or more concentrations of SR1 treated cells. Of those, 9 were differentially expressed in our dataset of cultured CD34+CD38−CD90+ cells (illustrated in heat map above). The heat map was generated on probe sets called as “Present” using the MAS5 algorithm in at least in one sample and if the absolute expression level was above 50. Several of these genes are known members of the AHR signaling pathway, which is a negative regulator of HSC function [1], [2]. Robust activation of this pathway was observed in CD34+CD38−CD90+ cells cultured for 12 hours, with restoration to almost normal levels by 2 weeks. AHRR negatively regulates AHR, however, it’s own transcription is regulated by AHR activation [3], [4]. CYP1B1 is also regulated by AHR activation [1]. Interestingly, the inflammatory cytokine, CCL2, has been linked to activation of mTOR [5], which compromises HSC function. Therefore, activation of AHR signaling and inflammatory pathways at the onset of ex vivo culture may trigger downstream effectors that negatively regulate CD34+CD38−CD90+ cell function. (TIF) [file pone.0053912.s008.tif]

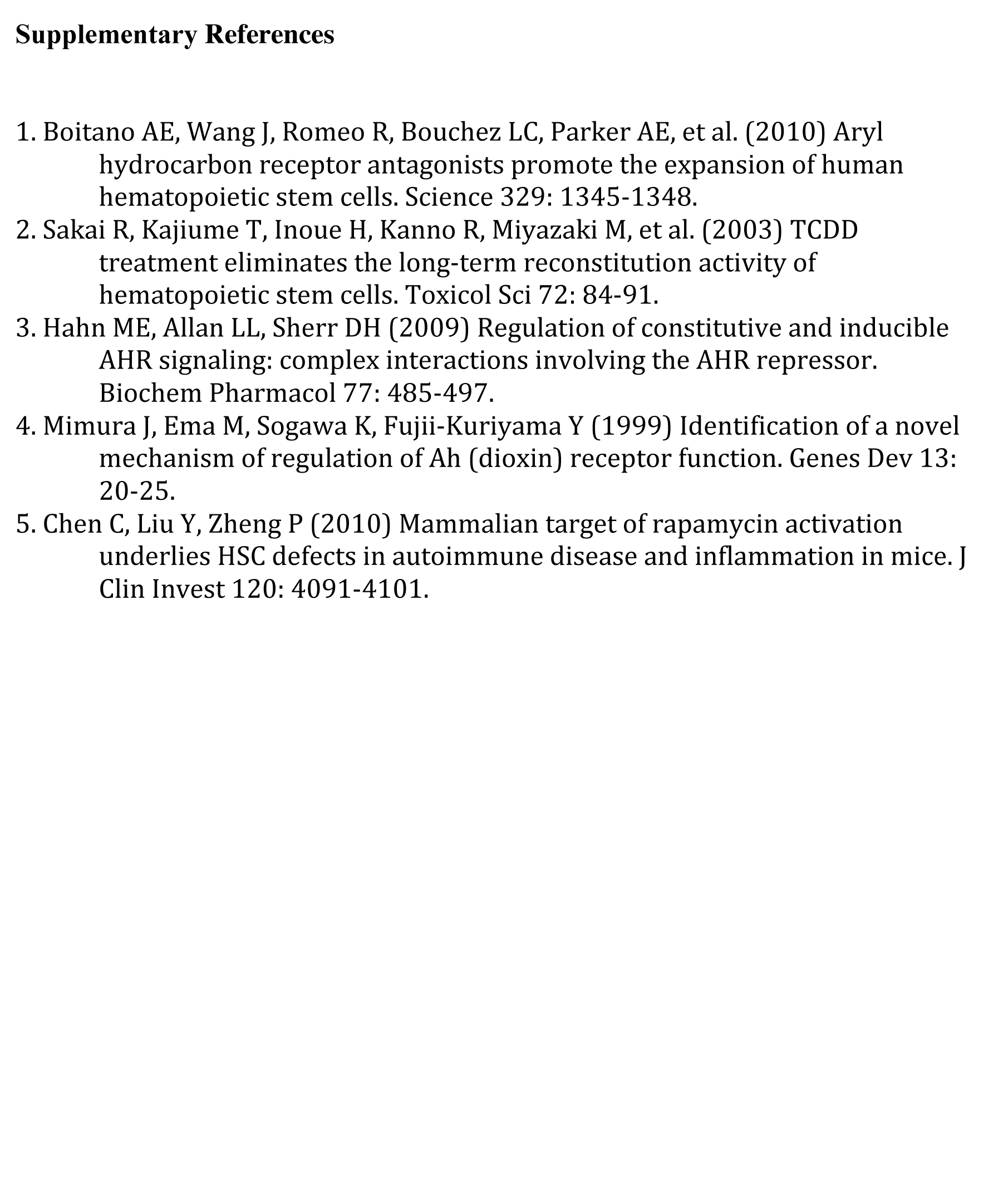

Supplement: References S1 — (TIF) [file pone.0053912.s012.tif]
